# Elevated homocysteine associated with higher cardiovascular risk in young patients with myocardial infarction: the role of metabolic phenotypes

**DOI:** 10.3389/fendo.2026.1821132

**Published:** 2026-05-08

**Authors:** Jing-yu Liu, Yu-hang Wang, Chang-ping Li, Zhuang Cui, Yin Liu, Jing Gao

**Affiliations:** 1 Clinical School of Thoracic, Tianjin Medical University, Tianjin, China; 2 Department of Cardiology, Tianjin Chest Hospital, Tianjin, China; 3 School of Public Health, Tianjin Medical University, Tianjin, China; 4 Chest Hospital, Tianjin University, Tianjin, China; 5 Cardiovascular Institute, Tianjin Chest Hospital, Tianjin, China; 6 Tianjin Key Laboratory of Cardiovascular Emergency and Critical Care, Tianjin, China

**Keywords:** homocysteine, metabolic syndrome, obesity, premature myocardial infarction, risk stratification

## Abstract

**Background:**

Premature myocardial infarction (PMI) is increasingly recognized as a metabolic disease, driven by a cluster of dysregulations including insulin resistance and central obesity. While homocysteine (Hcy)—an intermediate of one-carbon metabolism—is implicated in these atherothrombotic processes, its synergistic effect with coexisting cardiometabolic risk factors for the long-term prognosis of PMI patients remains unclear.

**Methods:**

We conducted a prospective cohort study of 1,220 consecutive PMI patients who underwent percutaneous coronary intervention (PCI) (men ≤45 years, women ≤55 years) at Tianjin Chest Hospital from January 2017 to December 2024. Plasma Hcy was measured at baseline and classified as high (≥15 µmol/L) or normal (<15 µmol/L). The primary endpoint was major adverse cardiovascular events (MACE), including cardiac death, rehospitalization for severe heart failure, non-fatal myocardial infarction, ischemic stroke, readmission for unstable angina, and target lesion revascularization. Multivariable Cox models and joint effect analyses were used to assess independent and synergistic associations.

**Results:**

Over a median follow-up of 582 days, 136 patients (11.1%) experienced MACE. After multivariable adjustment, high Hcy remained independently associated with MACE (aHR=2.72; 95% CI 1.87-3.95, P<0.001). This risk was higher when cardiometabolic factors coexisted, increasing from a 3.1-to 4.1-fold higher risk when combined with high D-Dimer, high-normal blood pressure, Lp(a), or hs-CRP (aHRs 3.13-4.12), to a 6.5-fold higher risk with coexisting obesity (aHR=6.54; 95% CI 3.01-14.20, P<0.001) compared to patients with normal Hcy and normal BMI. In the exploratory three-way analyses, the combination of high Hcy, obesity, and elevated D-Dimer (D-Dimer>0.5mg/L) conferred a nearly 10-fold higher risk (aHR=9.91; 95% CI 3.68–26.66, P<0.001), while the triad of high Hcy, obesity, and high hs-CRP (hs-CRP >2 mg/L) was associated with a 17.5-fold higher risk (aHR=17.51; 95% CI 2.28-134.52, P=0.006).

**Conclusion:**

Elevated plasma Hcy is an independent predictor of MACE in PMI patients and may be used to identify subgroups at particularly high risk. This effect is more pronounced in individuals who are obese or have other adverse metabolic characteristics.

## 1 Introduction

Premature myocardial infarction (PMI) patients have a considerable residual risk, which emphasizes the need for new prognostic biomarkers that reflect underlying pathophysiology (1). Metabolic Syndrome (MetS), defined as a cluster of metabolic abnormalities that consists of insulin resistance, central obesity, dyslipidemia, and hypertension (1, 2), has been proved to be the most frequently seen risk factor among patients presenting with young patients with coronary artery disease (CAD) (3). These metabolic abnormalities together induce the persistent, low-grade pro-inflammatory state known as “metaflammation” (1, 2). Also, hyperglycemia and hypercholesterolemia activate critical innate immune pathways by promoting myeloid-biased leukocytosis (4), generating a hyper-inflammatory memory that accelerates atherosclerosis at a young age (1, 2).

According to the previous investigations, homocysteine(Hcy) is regarded as a promising candidate. Hcy is an intermediate of one-carbon metabolism (5), a critical nutritional pathway linking methylation status to oxidative stress (6). Elevated Hcy has been associated with increased oxidative stress, reduced nitric oxide levels, and a prothrombotic and pro-inflammatory state (7, 8). Yet, its clinical narrative is riddled with contradictions. While observational data consistently link elevated Hcy to catastrophic cardiovascular outcomes (9–11), causality remains elusive. Mendelian randomization studies and failed B-vitamin randomized controlled trials (RCTs) (12) challenged this association. This discrepancy suggests a more complex reality: Hcy might be acting as a marker of residual confounding rather than an isolated causal factor (13, 14), which suggests that Hcy’s true pathogenicity may only be unveiled when stratified by metabolic status, rather than viewed in isolation.

Taking into account the unique metabolism of PMI, whether Hcy has an independent predictive role still needs to be confirmed in this population. Taking into account the results of previous research, it can be reasonably anticipated that there will be a considerable interactive effect among homocysteine (Hcy) and other risk factors. However, the specific pathway through which Hcy interacts with metabolic and inflammatory elements of PMI to influence long-term consequences is not yet clear. Therefore, we conducted this study.

## 2 Methods

### 2.1 Ethical conduct and data availability

All research protocols strictly complied with the ethics recommendations in the Declaration of Helsinki and obtained all necessary consents from the Institutional Review Board of Tianjin Chest Hospital (Approval No. 2017key-007-01). All participants signed a written informed consent form prior to enrollment, and the relevant procedures fully complied with ethical guidelines. In order to ensure transparency and also in accordance with the recommendations from the TOP guidelines, we are ready to make some supplementary materials available after reasonable contact inquiries.

### 2.2 Study population

**Figure 1:**
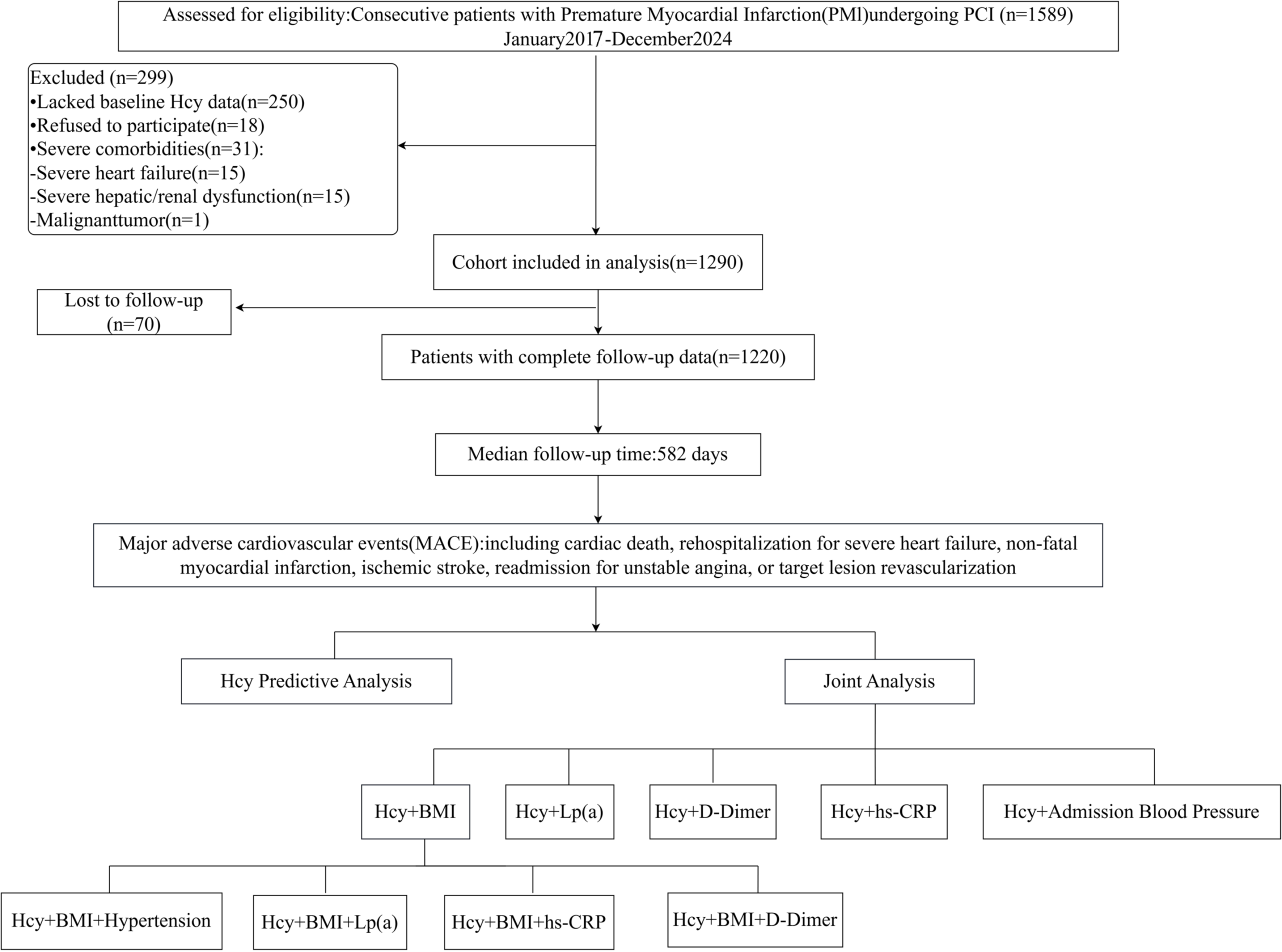
Flowchart of study population enrollment and analytical strategy. PMI, premature myocardial infarction; PCI, percutaneous coronary intervention; Hcy, homocysteine; MACE, major adverse cardiovascular events; BMI, body mass index; Lp(a), lipoprotein(a); hs-CRP, high-sensitivity C-reactive protein.

The final study population consisted of 1,220 PMI patients treated with percutaneous coronary intervention (PCI) at the Coronary Care Unit (CCU) of Tianjin Chest Hospital. Recruitment spanned from January 2017 to December 2024, drawing from an initial pool of 1,589 consecutive eligible patients. 299 participants were removed from the dataset following specific criteria, as detailed in the enrollment flow chart (**Figure 1**).

The inclusion criteria required that patients met the fourth universal definition of myocardial infarction and underwent PCI for PMI. Specifically, the diagnosis required fluctuating cardiac troponin (cTn) values—rising and/or falling with at least one measurement exceeding the 99th percentile upper reference limit. Furthermore, this must be associated with clinical indicators such as ischemia symptoms, new pathological Q waves, significant ECG changes (e.g., ST-T wave deviations or left bundle branch block), or imaging evidence of new loss of viable myocardium or regional wall motion abnormalities consistent with ischemia. Finally, an intracoronary thrombus via angiography or autopsy was also considered diagnostic. Age criteria for PMI were defined as ≤45 years for male patients and ≤55 years for female patients.

We removed participants if they presented with missing baseline Hcy data or significant structural heart disease (e.g., valvular or congenital heart disease). Also, we excluded individuals with pulmonary embolism or severe heart failure, defined as Killip class IV or a left ventricular ejection fraction (LVEF) ≤ 30%.Other exclusion rules included any cancer with a life expectancy of less than one year; advanced hepatic (Child-Pugh class C) or renal insufficiency, defined as estimated glomerular filtration rate (eGFR) < 30 mL/min/1.73 m².

### 2.3 Data collection and variable definitions

We collected baseline characteristics of each individual, such as age, gender, body mass index (BMI) smoking status and alcohol use. Past medical records were screened for major comorbidities, ranging from hypertension and diabetes to chronic kidney disease, cerebrovascular disease, angina, myocardial infarction, and other forms of CAD. Any previous revascularization procedures including coronary artery bypass grafting (CABG) or percutaneous coronary intervention (PCI) were recorded. All data mentioned above came from the electronic medical recording system of Tianjin Chest Hospital. At admission, key clinical indicators comprised vital signs, the eventual diagnosis (ST-segment elevation myocardial infarction [STEMI], or non-STEMI [NSTEMI]), information provided by angiography (the Gensini score, the number of diseased arteries, the existence of left main illness) and LVEF from echocardiography. Medication review focused on guideline-directed therapies, verifying the use of dual antiplatelet therapy (DAPT), statins, and angiotensin-converting enzyme inhibitors/angiotensin receptor blockers (ACEI/ARB).

Routine hematologic analysis assessed a complete blood count (leukocytes, neutrophil ratios, erythrocytes, hemoglobin, and platelets), as well as inflammatory markers like high-sensitivity C-reactive protein (hs-CRP) (15), and coagulation indicators such as D-Dimer and fibrinogen. The biochemical profile covered fasting plasma glucose (FPG), HbA1c, renal and liver function indicators, uric acid, total cholesterol (TC), triglycerides (TG), HDL-C, LDL-C, VLDL-C, lipoprotein(a) (Lp(a)), apolipoprotein A1 (ApoA1), and apolipoprotein B (ApoB); non-high-density lipoprotein cholesterol (Non-HDL-C) was calculated as TC minus HDL-C. To evaluate hemodynamic stress, we measured N-terminal pro-B-type natriuretic peptide (NT-proBNP). Myocardial injury indicators such as cardiac troponin T (cTnT), CK-MB, and creatine kinase (CK) were also tested.

Several important biomarkers were grouped for analytical purposes using evidence and established clinical recommendations. We adopted the BMI criteria tailored for Chinese adults: BMI was classified as normal weight (< 24.0 kg/m²) or overweight (24.0–27.9 kg/m²). Participants were considered obese if their BMI reached or exceeded 28.0 kg/m² (16). The American Diabetes Association (ADA) criteria were used to categorize the fasting glycemic status: normal (<5.6 mmol/L), impaired (5.6–6.9 mmol/L), or diabetes range (≥7.0 mmol/L) (17). While multiple lipid parameters including LDL-C were assessed, Lp(a) was specifically selected as the representative lipid marker for all subgroup analyses and interaction analyses. This choice was motivated by the distinct pathophysiology of Lp(a), which, unlike standard lipid particles, has innate prothrombotic and pro-inflammatory properties in addition to contributing to atherogenesis. Lp(a) was dichotomized at 50 mg/dL, a threshold suggested by the 2019 ESC/EAS guidelines to identify high lifetime cardiovascular risk. This stems from the fact that Lp(a) levels remain virtually unresponsive to conventional statin therapies, serving as a critical marker for the residual risk that endures even after LDL-C is optimally controlled (18). A cutoff of 0.5 mg/L was used to classify D-Dimer, a threshold widely recognized for its predictive value in acute coronary syndromes (19). Elevated hs-CRP was defined as a concentration > 2 mg/L (20). Finally, admission blood pressure was categorized into three groups based on systolic (SBP) and diastolic (DBP) measurements: normal (< 120/80 mmHg); high-normal (SBP 120–139 mmHg or DBP 80–89 mmHg); and high (≥140/90 mmHg) (21).

### 2.4 Homocysteine measurement

Following an 8- to 12-hour fast, fasting venous blood samples were taken from each patient in the morning within 24 hours after admission. Samples were gathered in tubes with EDTA and processed in an hour. Centrifugation (3000 rpm, 10 min, 4 °C) was performed to yield plasma, which was then promptly transferred to -80 °C storage for future preservation. A common enzymatic cycling test was used to determine plasma Hcy concentrations at the Department of Clinical Laboratory of Tianjin Chest Hospital. The diagnostic threshold for hyperhomocysteinemia (HHcy) was set at ≥15 µmol/L, according to the reference standard of Tianjin Chest Hospital.

### 2.5 Follow-up and clinical endpoints

Patients were tracked after discharge by trained investigators via clinic visits, telephone interviews, or the use of standard surveys. We maintained a median follow-up time of 582 days. MACE served as the primary outcome measure, which encompassed cardiac mortality, readmission due to severe heart failure, non-fatal myocardial infarction, ischemic stroke, hospitalization for unstable angina, and target lesion revascularization (TLR).TLR referred strictly to any ischemia-driven, any unplanned ischemia-driven revascularization of the treated lesion.

### 2.6 Statistical analysis

We conducted all data analyses using R software (v. 4.4.3) and Python (v. 3.11.7). The normality of continuous variables was assessed using the Kolmogorov-Smirnov test. As all continuous variables in this study were found to be non-normally distributed, they were presented as medians with interquartile range (IQR, 25th–75th percentiles) to describe central tendency and variation, respectively.

For comparative analysis between groups, the Mann-Whitney U test was used for two independent groups (e.g., MACE vs. No MACE), and the Kruskal-Wallis test was employed for comparisons involving three or more groups. Categorical variables were expressed as counts and percentages (n, %) and were compared using the Chi-square test or Fisher’s exact test (if expected cell counts were < 5).

Kaplan-Meier plot were used to present time-to-event data, and the log-rank test was used to compare differences between groups. Both univariate and multivariate Cox proportional-hazards models were used; HRs and their corresponding 95% CIs were obtained to assess the prognostic significance of Hcy and other factors. To investigate the possible non-linear relationship between continuous Hcy levels and MACE risk, restricted cubic splines (RCS) were performed. We also conducted subgroup analyses stratified by key covariates. By adding a cross-product term (such as Hcy category × BMI category) to the fully adjusted Cox models, we officially tested for multiplicative interactions and provided the appropriate P value for interaction. We conducted a sensitivity analysis by computing the E-value for the adjusted hazard ratio of the primary exposure (high Hcy) in order to assess the robustness of our main finding against any unmeasured confounding.

Missing data accounted for <5% of all baseline variables. Five imputed datasets were created for the analysis by utilizing the multiple imputation by chained equations (MICE) package in R to address missing variables. During follow-up, 70 participants (5.4%) dropped out or could not be reached; they were right-censored at their last known follow-up date. To assess potential attrition bias, we compared the baseline characteristics of patients lost to follow-up with those who completed the study (**Supplementary Table 1**).

We deemed results to be statistically significant only if the two-sided P-value fell below 0.05. This manuscript was drafted in strict accordance with the guidelines outlined in the STROBE (Strengthening the Reporting of Observational Studies in Epidemiology) statement.

## 3 Results

### 3.1 Baseline characteristics and clinical events

The study analyzed 1,220 PMI patients. MACE occurred in 136 individuals (11.1%) during a median follow-up period of 582 days. The components of these adverse events consisted of 17 cardiac deaths, 9 ischemic strokes, 19 nonfatal myocardial infarctions, along with 35 readmissions for severe heart failure, 38 target lesion revascularizations and 18 readmissions for unstable angina.

**Table 1** provides a comprehensive comparison of baseline features, categorized according to whether patients experienced MACE. Male was the predominant gender in the cohort (1082/1220, 88.69%), and the participants’ median age was 41 years (interquartile range [IQR], 37.00-44.00). Broadly, the two subgroups shared similar demographic and clinical backgrounds, except for some variations in specific parameters. Inflammatory indicators such as white blood cell (WBC) (median 10.79 vs. 10.10×10^9^/L, P = 0.005) and hs-CRP (median 6.69 vs. 5.19 mg/L, P = 0.004), were greater in patients of MACE group. Likewise, a far higher percentage of patients in the MACE group had increased hs-CRP levels (>2 mg/L)(89.71% vs. 76.94%, P < 0.001). The admission values of CK-MB (median 120 vs. 81 U/L, P < 0.001), cTnT(median 3.31 vs. 1.71 ng/mL, P < 0.001), and NT-proBNP(median 270 vs. 149 pg/mL, P = 0.003) were considerably higher, while LVEF much lower(median 50% vs. 53%, P < 0.001) in the MACE group, which meant worsened cardiac function and more severe myocardial damage.

**Table 1:** Baseline characteristics of patients stratified by MACE.

Variables	Total population (N = 1220)	No MACE (N = 1084)	MACE (N = 136)	P-value
Baseline demographics				
Age (years)	41.00 (37.00–44.00)	41.00 (37.00–44.00)	41.00 (36.00–44.00)	0.314
Male, n (%)	1,082 (88.69)	957 (88.28)	125 (91.91)	0.208
BMI (kg/m²)	26.00 (23.90–28.10)	26.00 (23.90–28.10)	26.10 (24.20–28.70)	0.505
BMI category, n (%)				0.388
< 24.0 kg/m²	308 (25.25)	278 (25.65)	30 (22.06)	
24.0–27.9 kg/m²	591 (48.44)	527 (48.62)	64 (47.06)	
≥ 28.0 kg/m²	321 (26.31)	279 (25.74)	42 (30.88)	
Admission blood pressure, n (%)^a^				0.521
Normal	240 (19.67)	216 (19.93)	24 (17.65)	
High-normal	461 (37.79)	413 (38.10)	48 (35.29)	
High	519 (42.54)	455 (41.97)	64 (47.06)	
History of smoking, n (%)	761 (62.38)	675 (62.27)	86 (63.24)	0.827
History of alcohol consumption, n (%)	398 (32.62)	355 (32.75)	43 (31.62)	0.791
Medical history				
History of hypertension, n (%)	586 (48.03)	517 (47.69)	69 (50.74)	0.503
History of diabetes, n (%)	252 (20.66)	223 (20.57)	29 (21.32)	0.838
History of chronic kidney disease, n (%)	15 (1.23)	14 (1.29)	1 (0.74)	>0.999
History of cerebrovascular disease, n (%)	41 (3.36)	35 (3.23)	6 (4.41)	0.448
Prior angina, n (%)	170 (13.93)	155 (14.30)	15 (11.03)	0.299
Prior myocardial infarction, n (%)	68 (5.57)	57 (5.26)	11 (8.09)	0.175
Prior coronary artery disease, n (%)	126 (10.33)	112 (10.33)	14 (10.29)	0.989
Prior PCI, n (%)	47 (3.85)	41 (3.78)	6 (4.41)	0.719
Prior CABG, n (%)	8 (0.66)	7 (0.65)	1 (0.74)	>0.999
Blood routine and inflammatory indicators				
WBC (10^9^/L)	10.17 (8.38–12.30)	10.10 (8.30–12.19)	10.79 (9.14–12.61)	0.005
NEUT (%)	73.50 (66.50–79.90)	73.20 (66.20–79.70)	75.86 (69.65–80.95)	0.010
RBC (10¹²/L)	4.85 (4.49–5.18)	4.84 (4.48–5.17)	4.93 (4.52–5.22)	0.210
Hb (g/L)	147 (137–157)	147 (137–156)	150 (139–160)	0.070
PLT (10^9^/L)	241 (208–283)	241 (208–282)	240 (207–286)	0.993
hs-CRP (mg/L)	5.34 (2.38–11.55)	5.19 (2.21–11.45)	6.69 (3.33–14.66)	0.004
hs-CRP category, n (%)				<0.001
≤2 mg/L	264 (21.64)	250 (23.06)	14 (10.29)	
> 2mg/L	956 (78.36)	834 (76.94)	122 (89.71)	
Biochemical parameters				
HbA1c (%)	5.80 (5.40–7.20)	5.80 (5.40–7.00)	5.80 (5.40–8.00)	0.381
FPG (mmol/L)	5.77 (5.06–7.51)	5.71 (5.05–7.46)	6.24 (5.09–8.48)	0.017
FPG category, n (%)				0.035
<5.6mmol/L	534 (43.77)	488 (45.02)	46 (33.82)	
5.6–6.9mmol/L	321 (26.31)	282 (26.01)	39 (28.68)	
≥28.mmol/L	365 (29.92)	314 (28.97)	51 (37.50)	
BUN (mmol/L)	4.30 (3.60–5.30)	4.30 (3.60–5.30)	4.60 (3.70–5.40)	0.195
Cr (μmol/L)	73 (64–83)	73 (63–82)	77 (68–89)	<0.001
Uric acid (μmol/L)	356 (296–427)	356 (295–424)	365 (308–452)	0.064
TBIL (μmol/L)	13.96 (10.00–19.00)	13.72 (9.82–18.80)	15.40 (10.98–21.60)	0.009
DBIL (μmol/L)	4.20 (2.60–5.90)	4.10 (2.41–5.70)	5.15 (3.90–6.85)	<0.001
ALT (U/L)	42.35 (27.20–68.05)	42.20 (26.40–67.70)	43.60 (31.90–74.85)	0.063
AST (U/L)	103.60 (42.35–208.30)	98.20 (39.80–204.30)	137.55 (70.20–244.15)	<0.001
ALP (U/L)	75 (63–90)	75 (63–90)	72 (63–89)	0.329
GGT (U/L)	37.80 (25.00–57.05)	37.00 (25.00–56.35)	41.80 (27.05–61.50)	0.088
Hcy (μmol/L)	12.30 (9.61–16.85)	12.07 (9.50–15.90)	15.75 (10.57–28.97)	<0.001
Hcy category, n (%)				<0.001
<15 μmol/L	832 (68.20)	774 (71.40)	58 (42.65)	
≥15μmol/L	388 (31.80)	310 (28.60)	78 (57.35)	
Lipid profile				
Lp(a) (mg/dL)	72.96 (22.55–192.13)	76.22 (22.55–199.01)	61.20 (22.60–154.05)	0.176
Lp(a) category, n (%)				0.370
<50 mg/dL	486 (39.84)	427 (39.39)	59 (43.38)	
≥50mg/dL	734 (60.16)	657 (60.61)	77 (56.62)	
TC (mmol/L)	4.86 (4.14–5.56)	4.84 (4.12–5.53)	4.96 (4.24–5.75)	0.261
TG (mmol/L)	2.03 (1.45–3.03)	2.03 (1.44–3.05)	2.06 (1.48–2.84)	0.869
HDL-C (mmol/L)	0.92 (0.82–1.06)	0.92 (0.83–1.06)	0.92 (0.79–1.07)	0.198
LDL-C (mmol/L)	3.18 (2.47–3.80)	3.17 (2.46–3.77)	3.20 (2.64–4.02)	0.089
VLDL-C (mmol/L)	0.58 (0.38–0.85)	0.59 (0.38–0.85)	0.55 (0.39–0.79)	0.537
ApoA1 (g/L)	1.13 (1.00–1.26)	1.13 (1.00–1.26)	1.11 (0.99–1.26)	0.454
ApoB (g/L)	1.14 (0.93–1.35)	1.14 (0.93–1.34)	1.19 (1.01–1.43)	0.058
Non-HDL-C (mmol/L)	3.89 (3.16–4.60)	3.90 (3.14–4.58)	3.86 (3.23–4.85)	0.377
Myocardial injury markers				
CK (U/L)	987 (324–2,073)	924 (299–1,999)	1,401 (641–2,340)	0.002
CK-MB (U/L)	84 (30–176)	81 (28–174)	120 (54–197)	<0.001
cTnT (ng/mL)	1.88 (0.50–4.29)	1.71 (0.42–4.13)	3.31 (1.30–5.84)	<0.001
NT-proBNP (pg/mL)	153 (29–473)	149 (26–438)	270 (38–808)	0.003
LVEF (%)	53 (47–57)	53 (47–57)	50 (42–56)	<0.001
Coagulation				
D-Dimer (mg/L)	0.26 (0.19–0.42)	0.26 (0.19–0.41)	0.29 (0.20–0.51)	0.069
D-Dimer category, n (%)				0.006
≤0.5 mg/L	1,001 (82.05)	901 (83.12)	100 (73.53)	
>0.5 mg/L	219 (17.95)	183 (16.88)	36 (26.47)	
Fibrinogen (g/L)	3.32 (2.86–3.93)	3.31 (2.85–3.93)	3.37 (2.89–3.95)	0.259
Clinical and angiographic features				
Diagnosis, n (%)				0.003
STEMI	936 (76.72)	818 (75.46)	118 (86.76)	
NSTEMI	284 (23.28)	266 (24.54)	18 (13.24)	
Number of diseased vessels, n (%)				0.441
No significant stenosis	41 (3.36)	34 (3.14)	7 (5.15)	
Single-vessel disease	472 (38.69)	426 (39.30)	46 (33.82)	
Double-vessel disease	326 (26.72)	291 (26.85)	35 (25.74)	
Triple-vessel disease	352 (28.85)	308 (28.41)	44 (32.35)	
Left main disease	29 (2.38)	25 (2.31)	4 (2.94)	
Gensini score	26 (0–52)	26 (0–52)	23 (0–48)	0.222
Concomitant medications				
DAPT, n (%)	1,215 (99.59)	1,080 (99.63)	135 (99.26)	0.447
Statins, n (%)	1,192 (97.70)	1,061 (97.88)	131 (96.32)	0.229
ACEI/ARB, n (%)	806 (66.07)	706 (65.13)	100 (73.53)	0.051
Beta-blocker, n (%)	944 (77.38)	830 (76.57)	114 (83.82)	0.057

^a^ Admission blood pressure: Normal: the systolic blood pressure (SBP) <120 mmHg and the diastolic blood pressure (DBP) is <80 mmHg; Normal-High: the SBP is between 120 to139 mmHg and/or the DBP is between 80 to 89 mmHg; High: the SBP ≥140 mmHg and/or the DBP ≥90 mmHg. ACEI, angiotensin-converting enzyme inhibitor; ALT, alanine aminotransferase; ApoA1, apolipoprotein A1; ApoB, apolipoprotein B; ARB, angiotensin receptor blocker; AST, aspartate aminotransferase; BMI, body mass index; BUN, blood urea nitrogen; CABG, coronary artery bypass grafting; CK, creatine kinase; CK-MB, creatine kinase-MB; Cr, creatinine; cTnT, cardiac troponin T; DAPT, dual antiplatelet therapy; DBIL, direct bilirubin; FPG, fasting plasma glucose; GGT, gamma-glutamyl transferase; Hb, hemoglobin; HbA1c, glycated hemoglobin A1c; Hcy, homocysteine; HDL-C, high-density lipoprotein cholesterol; hs-CRP, high-sensitivity C-reactive protein; LDL-C, low-density lipoprotein cholesterol; Lp(a), lipoprotein(a); LVEF, left ventricular ejection fraction; MACE, major adverse cardiovascular events; NEUT, neutrophil; Non-HDL-C, non-high-density lipoprotein cholesterol; NSTEMI, non-ST-segment elevation myocardial infarction; NT-proBNP, N-terminal pro-B-type natriuretic peptide; PCI, percutaneous coronary intervention; PLT, platelet; RBC, red blood cell; STEMI, ST-segment elevation myocardial infarction; TBIL, total bilirubin; TC, total cholesterol; TG, triglyceride; VLDL-C, very-low-density lipoprotein cholesterol; WBC, white blood cell.

Importantly, we observed a dramatical difference in Hcy profiles between the cohorts. The median was significantly elevated (15.75 vs. 12.07 μmol/L; P <0.001) in those who experienced MACE (n=136) compared to their counterparts (n=1,084). 57.35% of patients in the MACE group had elevated Hcy (≥15 μmol/L),while in the non-MACE group the frequency is 28.60% (P <0.001).

### 3.2 Independent prognostic value of Hcy

#### 3.2.1 Univariable predictors of MACE

Univariable Cox regression analysis was performed on a thorough panel of baseline variables to identify predictors of MACE (**Supplementary Figure 1**). The result revealed a statistical association between Hcy levels and the occurrence of MACE. Other significant univariable predictors were found in several pathophysiological domains, encompassing metabolic factors (e.g., BMI and FPG), markers of inflammation and thrombosis (e.g., WBC, hs-CRP, Lp(a), D-Dimer), and myocardial injury (e.g., cTnT, NT-proBNP) (P values ranged from <0.001 to 0.042, **Supplementary Figure 1**). Neither the history of diabetes (P = 0.983) nor hypertension (P = 0.820) was significantly associated with the primary endpoint.

#### 3.2.2 Independent prognostic value of Hcy

We subsequently developed multivariable Cox regression models to determine whether Hcy held independent prognostic value (**Table 2**). The correlation between high Hcy and MACE remained statistically significant even after controlling for demographics, conventional risk variables, and other important biomarkers (aHR=2.72; 95%CI 1.87–3.95, P<0.001). Similarly, when assessing Hcy on a continuous scale, its hazard ratio remained stable at 1.06 across all models (Crude Model: aHR=1.06; 95% CI: 1.04–1.07, P < 0.001; Model 1 aHR=1.06; 95% CI: 1.04–1.07, P < 0.001; Model 2: aHR=1.06; 95% CI: 1.04–1.07, P < 0.001; Model3: aHR=1.06; 95% CI: 1.05–1.08, P < 0.001).To further clarify the clinical significance of the composite endpoint, we conducted separate analyses of each component of MACE (**Table 3**). The results showed that higher Hcy levels were associated with an increased risk of multiple MACE component events. Specifically, Hcy was associated with non-fatal myocardial infarction (HR 5.86, 95% CI 2.10–16.30, P < 0.001), hospitalization for unstable angina (HR 3.40, 95% CI 1.32–8.76, P = 0.012), readmission for severe heart failure (HR 2.42, 95% CI 1.25–4.71, P = 0.009) and revascularization of the target lesion (TLR) (HR 2.07, 95% CI 1.10–3.91, P = 0.025). Cardiac death and stroke also showed higher HR values in the high Hcy group, but the differences did not reach statistical significance.

**Table 2:** Association between Hcy levels and risk of MACE in Cox regression models.

Characteristic		Crude model	Model 1	Model 2	Model 3
Hcy	HR (95% CI)	1.06 (1.04-1.07)	1.06 (1.04-1.07)	1.06 (1.04-1.07)	1.06 (1.05-1.08)
	P-value	<0.001	<0.001	<0.001	<0.001
Hcy (categorical)	Group 1	Ref	Ref	Ref	Ref
	Group 2	2.93 (2.09-4.12)	2.85 (2.01-4.04)	3.14 (2.19-4.50)	2.72 (1.87-3.95)
	Group 2 P-value	<0.001	<0.001	<0.001	<0.001

Hcy, homocysteine; MACE, major adverse cardiovascular events; HR, hazard ratio; CI, confidence interval; Ref, reference.

Model 1: Adjusted for age and sex.

Model 2: Adjusted for variables in Model 1 plus history of hypertension, history of diabetes, BMI, and admission blood pressure.

Model 3: Adjusted for variables in Model 2 plus WBC, hs-CRP, Lp(a), FPG, Cr, CK-MB, cTnT, NT-proBNP, D-Dimer, DAPT, statins, ACEI/ARB, and Beta-blockers.

**Table 3:** Individual component analysis of MACE by Hcy group.

Outcome	Total events	Low Hcy n (%)	High Hcy n (%)	HR (95% CI)	P value
Cardiac death	17	8 (1%)	9 (2.3%)	2.36 (0.91–6.12)	0.077
Nonfatal MI	19	5 (0.6%)	14 (3.6%)	5.86 (2.1–16.3)	<0.001
Stroke	9	4 (0.5%)	5 (1.3%)	2.64 (0.71–9.82)	0.149
Severe heart failure readmission	35	16 (1.9%)	19 (4.9%)	2.42 (1.25–4.71)	0.009
TLR	38	19 (2.3%)	19 (4.9%)	2.07 (1.1–3.91)	0.025
Unstable angina hospitalization	18	7 (0.8%)	11 (2.8%)	3.4 (1.32–8.76)	0.012

Hcy, homocysteine; MACE, major adverse cardiovascular events; HR, hazard ratio; CI, confidence interval; TLR, target lesion revascularization. HRs and 95% CIs were estimated using unadjusted Cox proportional hazards models.

### 3.3 Quantifying the combined prognostic impact of Hcy

#### 3.3.1 Joint prognostic impact of Hcy with cardiometabolic factors

**Figure 2:**
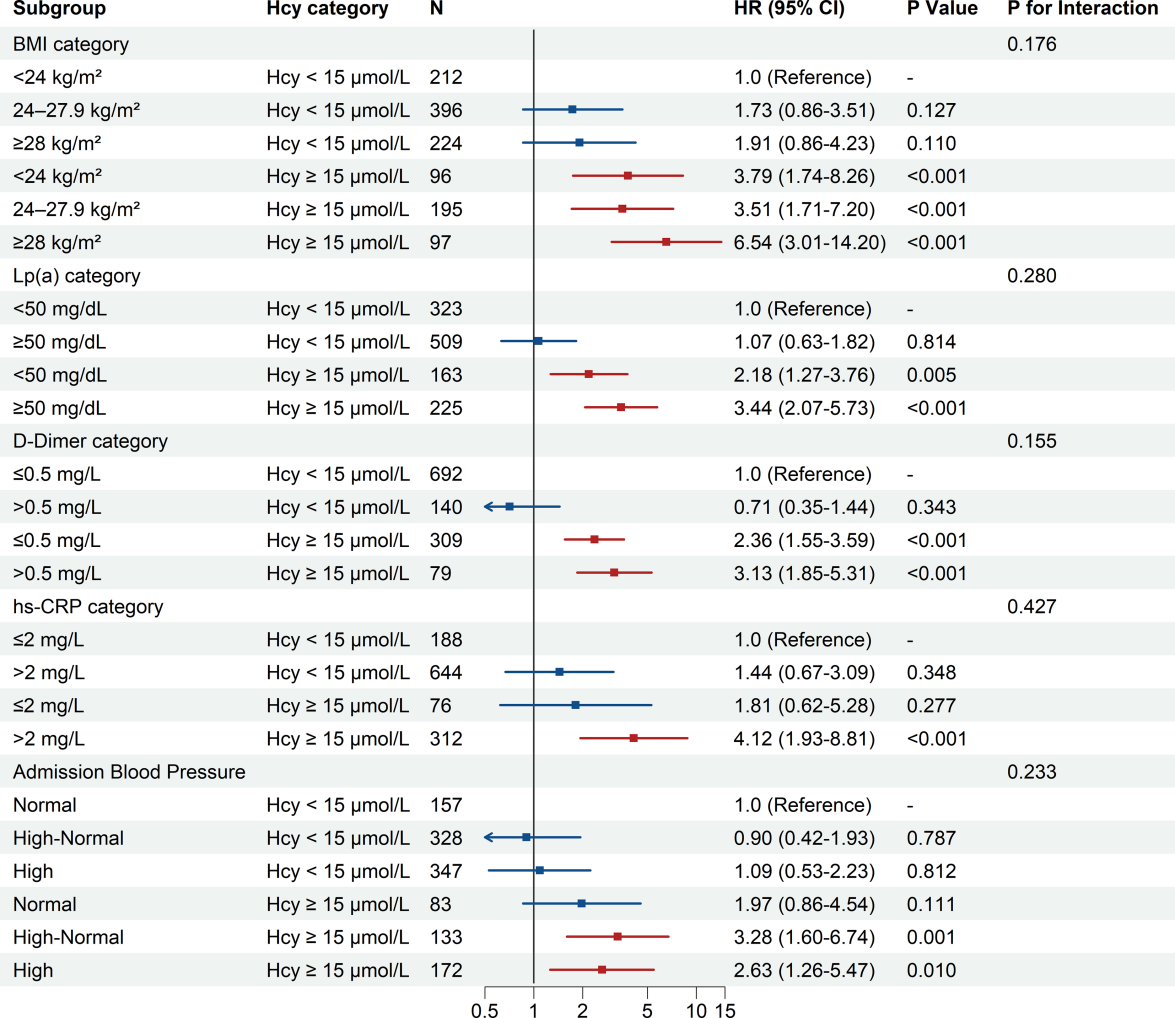
Forest plot of joint associations of homocysteine and cardiometabolic risk factors with major adverse cardiovascular events (MACE). Hazard ratios (HRs) and 95% confidence intervals (CIs) were derived from multivariable Cox proportional hazards models, assessing the combined prognostic value of homocysteine (Hcy) categories with body mass index (BMI), Lipoprotein(a), D-Dimer, high-sensitivity C-reactive protein (hs-CRP), and admission blood pressure. The reference group for each analysis was patients with Hcy < 15 µmol/L within the lowest-risk subgroup of the respective factor. Models were adjusted for age, sex, history of hypertension, history of diabetes, BMI, admission blood pressure, WBC, hs-CRP, Lp(a), FPG, Cr, CK-MB, cTnT, NT-proBNP, D-Dimer, DAPT, statins, ACEI/ARB, and Beta-blockers. The P for interaction was calculated to test for a multiplicative interaction between Hcy and each subgroup variable. ACEI/ARB, Angiotensin-Converting Enzyme Inhibitors/Angiotensin II Receptor Blockers; BMI, body mass index; CI, confidence interval; CK-MB, Creatine Kinase-MB isoenzyme; Cr, Creatinine; cTnT, Cardiac Troponin T; DAPT, Dual Antiplatelet Therapy; FPG, Fasting Plasma Glucose; Hcy, homocysteine; HR, hazard ratio; hs-CRP, high-sensitivity C-reactive protein; Lp(a), Lipoprotein(a); MACE, major adverse cardiovascular events; NT-proBNP, N-terminal pro-B-type Natriuretic Peptide; WBC, White Blood Cell count. Definitions for the clinical subgroups are provided in the legend of **Table 1**.

To quantify the joint prognostic impact of Hcy with other cardiometabolic risk factors, stratified multivariable Cox regression analyses were performed (**Figure 2**). Regardless of BMI category, participants with high Hcy levels exhibited higher MACE risk. Relative to participants with normal Hcy and normal BMI, the risk was elevated in the high Hcy/normal weight subgroup (aHR=3.79; 95% CI 1.74–8.26;P < 0.001) and the high Hcy/overweight subgroup (aHR=3.51; 95% CI 1.71–7.20;P < 0.001), and peaked in the high Hcy/obese subgroup (aHR=6.54; 95% CI 3.01–14.20; P < 0.001).

Similarly, participants with both elevated Hcy levels and other high-risk markers had a higher risk of MACE, with the highest risk observed among those with high hs-CRP (aHR=4.12; 95% CI: 1.93–8.81, P <0.001), high Lp(a) (aHR=3.44; 95% CI: 2.07–5.73, P <0.001), and high D-Dimer (aHR=3.13; 95% CI: 1.85–5.31, P <0.001). However, risks remained elevated in high Hcy patients with low D-Dimer levels (aHR=2.36; 95% CI: 1.55–3.59, P < 0.001) or low Lp(a) (aHR=2.18; 95% CI: 1.27–3.76, P = 0.005). When stratifying patients according to their blood pressure at admission, the high Hcy/High-Normal BP subgroup had the highest risk(aHR=3.28; 95% CI: 1.60–6.74, P = 0.001), followed by the HHcy/High blood pressure subgroup (aHR=2.63; 95% CI: 1.26–5.47, P = 0.010) (**Figure 2**).

**Figure 3:**
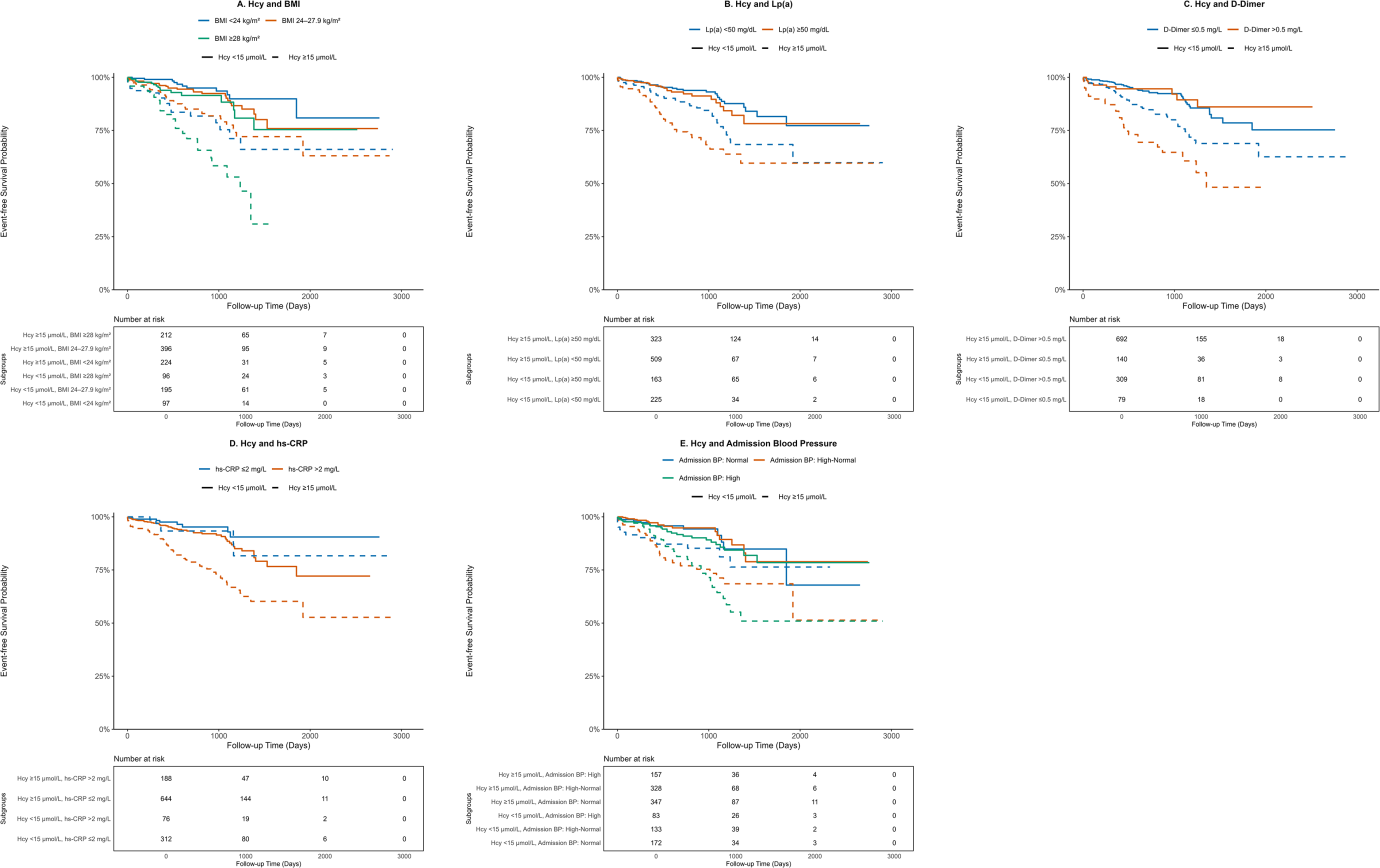
Kaplan-Meier curves for the joint effect of homocysteine and other risk factors on MACE-free survival. Kaplan-Meier curves showing MACE-free survival stratified by the joint effect of homocysteine levels and: **(A)** adiposity (stratified by BMI categories); **(B)** Lp(a) category; **(C)** prothrombotic state (stratified by D-Dimer); **(D)** inflammatory status (stratified by hs-CRP); and **(E)** admission blood pressure. Definitions for all clinical subgroups are provided in the legend of **Table 1**.

According to the Kaplan-Meier curves, the subgroup of patients with both high Hcy and the corresponding high-risk phenotype consistently exhibited the lowest MACE-free survival rate (log-rank P <0.001 for all comparisons, **Figure 3**).

**Figure 4:**
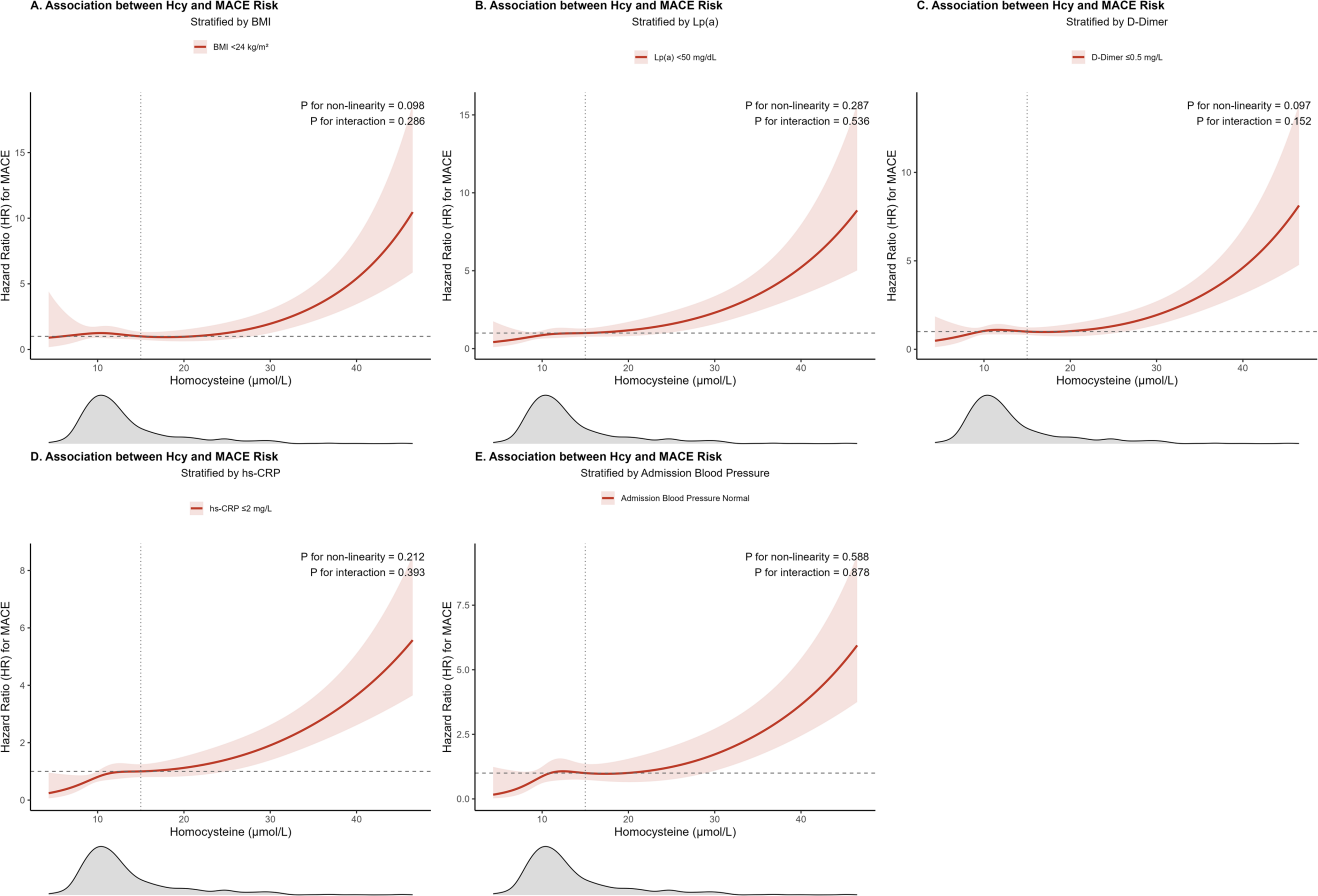
Dose-response relationship between homocysteine and MACE risk, stratified by key risk factors. Restricted cubic spline curves illustrating the dose-response relationship between homocysteine (Hcy) levels and the adjusted Hazard Ratio (aHR) for MACE within specific clinical subgroups: **(A)** patients with overweight; **(B)** the high lipoprotein(a) (Lp(a)) subgroup; **(C)** patients with a normal prothrombotic state (normal D-Dimer); **(D)** patients with a high inflammatory state (high hs-CRP); and **(E)** the high-normal admission blood pressure subgroup. Definitions for all clinical subgroups are provided in the legend of **Table 1**.

We employed restricted cubic spline models to characterize the dose-response relationship between continuous Hcy levels and MACE risk within specific strata (**Figure 4**). These analyses showed a generally increasing risk of MACE with higher Hcy levels. Formal tests for multiplicative interaction between Hcy and each of the partner variables were not statistically significant (P for interaction > 0.05).

#### 3.3.2 Exploratory three-way interaction analyses

We incorporated a third clinical parameter into the Hcy-BMI model to explore the high risk subgroups.

##### 3.3.2.1 Combined effect with hypertension

**Figure 5:**
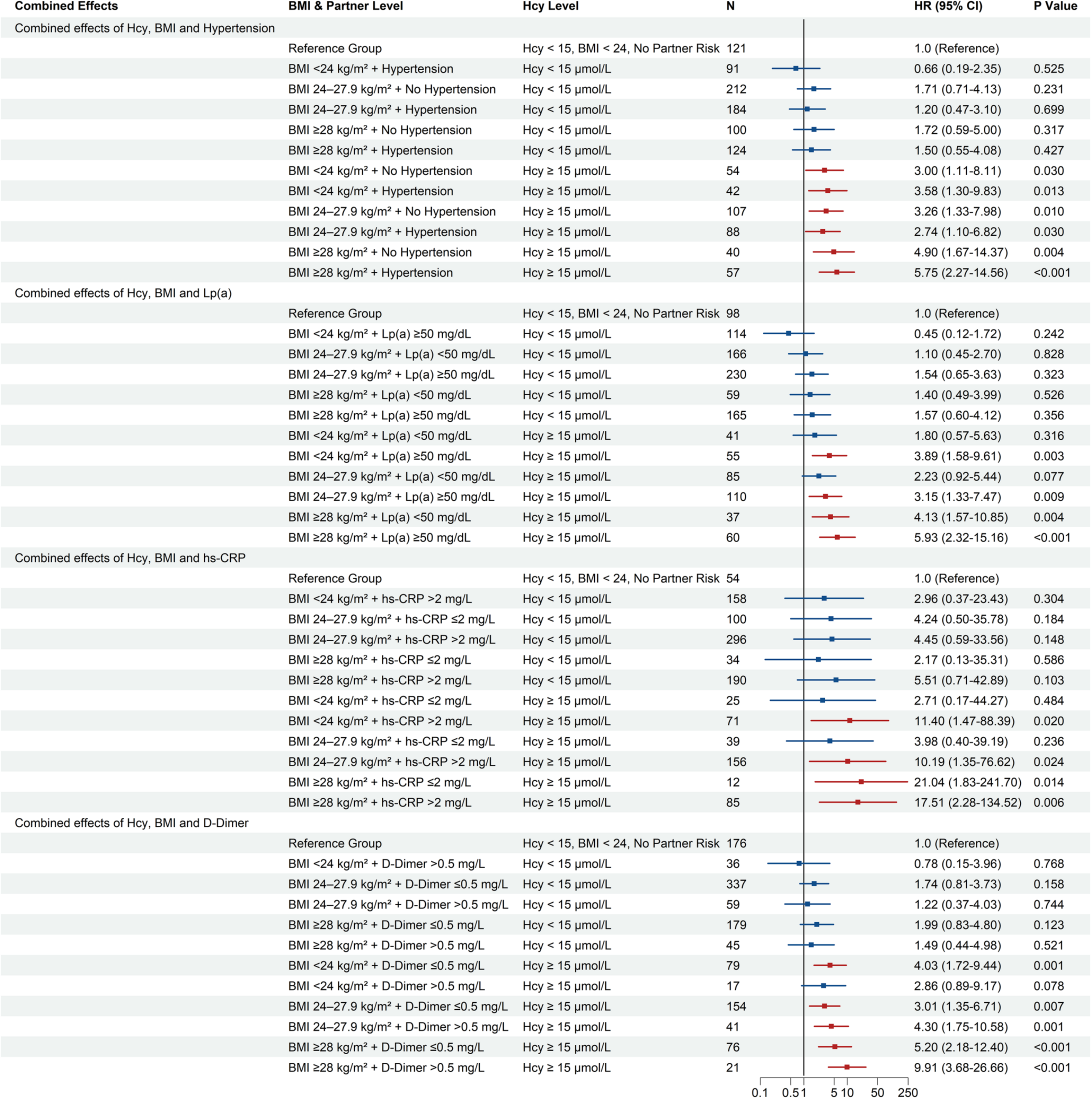
Forest plot of the combined prognostic effects of homocysteine, body mass index, and additional risk factors on major adverse cardiovascular events (MACE). Hazard ratios (HRs) and 95% confidence intervals (CIs) were derived from multivariable Cox proportional hazards models. The figure displays four separate analyses assessing the combined risk associated with various combinations of homocysteine (Hcy), body mass index (BMI), and a third partner risk factor: hypertension, Lipoprotein(a) (Lp(a)), high-sensitivity C-reactive protein (hs-CRP), or D-Dimer. For each of the four analyses, the reference group consisted of patients with the lowest-risk profile: Hcy <no><</no> 15 µmol/L, BMI <no><</no> 24 kg/m², and the absence of the third risk factor (i.e., no hypertension, Lp(a) ≤50 mg/dL, hs-CRP ≤2 mg/L, or D-Dimer ≤0.5 mg/L). Models were adjusted for age, sex, history of hypertension, history of diabetes, BMI, admission blood pressure, WBC, hs-CRP, Lp(a), FPG, Cr, CK-MB, cTnT, NT-proBNP, D-Dimer, DAPT, statins, ACEI/ARB, and Beta-blockers. ACEI/ARB, Angiotensin-Converting Enzyme Inhibitors/Angiotensin II Receptor Blockers; BMI, body mass index; CI, confidence interval; CK-MB, Creatine Kinase-MB isoenzyme; Cr, Creatinine; cTnT, Cardiac Troponin T; DAPT, Dual Antiplatelet Therapy; FPG, Fasting Plasma Glucose; Hcy, homocysteine; HR, hazard ratio; hs-CRP, high-sensitivity C-reactive protein; Lp(a), Lipoprotein(a); MACE, major adverse cardiovascular events; NT-proBNP, N-terminal pro-B-type Natrioturetic Peptide; WBC, White Blood Cell count.

**Figure 6:**
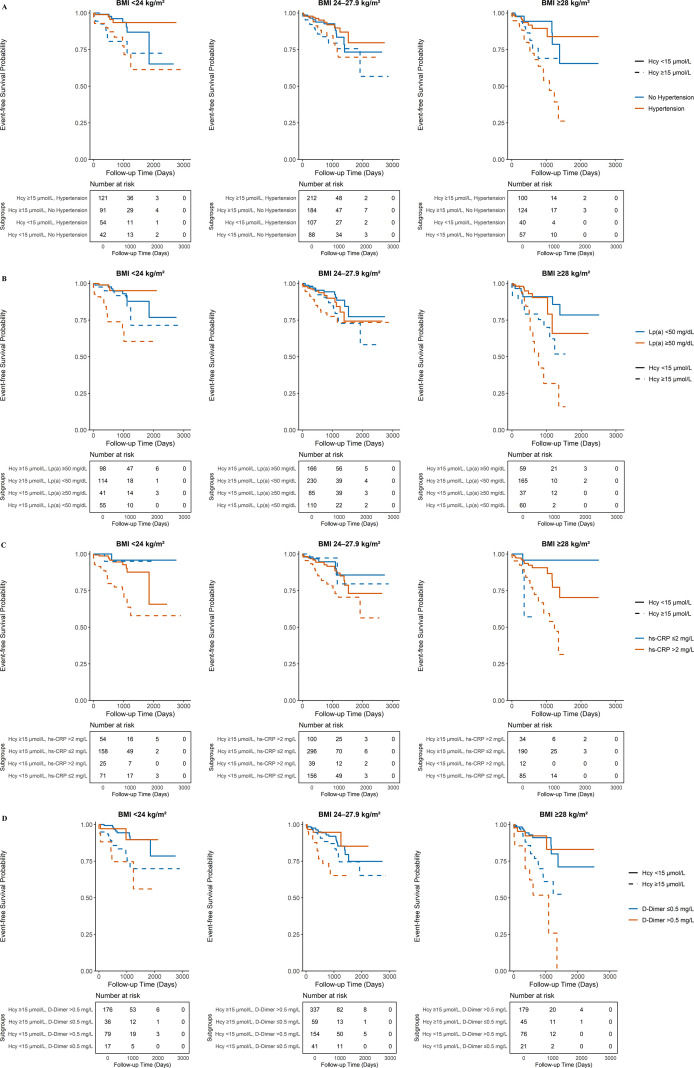
Kaplan-Meier curves for the three-way joint effect of Hcy, BMI, and a third risk factor on MACE-free survival. Kaplan-Meier curves showing MACE-free survival, stratified by the three-way joint effects of homocysteine (Hcy) levels, Body Mass Index (BMI), and: **(A)** a clinical history of hypertension; **(B)** lipoprotein(a) (Lp(a)) category; **(C)** inflammatory status (stratified by hs-CRP); and **(D)** prothrombotic state (stratified by D-Dimer).

The Hcy-BMI-Hypertension model showed progressively higher risks of MACE across subgroups (**Figure 5**). The highest risk was observed in patients with all three factors (obesity [BMI ≥28 kg/m²], high Hcy [≥15 µmol/L], and hypertension), representing a 5.75-fold higher MACE susceptibility when measured against the reference group (aHR=5.75; 95% CI: 2.27–14.56, P < 0.001). The Kaplan-Meier survival curves supported this risk stratification (**Figure 6A**).

##### 3.3.2.2 Combined effect with lipoprotein(a)

A similar risk profile was observed when combining Hcy, BMI, and Lp(a) categories (**Figure 5**). The subgroup with obesity (BMI ≥28 kg/m²), high Hcy (≥15 µmol/L), and high Lp(a) (≥50 mg/dL) had a 5.93-fold surge in MACE probability (aHR=5.93; 95% CI: 2.32–15.16, P < 0.001). The Kaplan-Meier survival curves’ separation was in line with this pattern (**Figure 6B**).

##### 3.3.2.3 Hazard ratios in the hs-CRP model

The three-way analysis involving hs-CRP had high hazard ratios with wide confidence intervals in some subgroups (**Figure 5**). The subgroup of obese (BMI ≥28 kg/m²), high-Hcy (≥15 µmol/L) patients with normal hs-CRP (≤2 mg/L) had an aHR of 21.04 (95% CI: 1.83–241.70, P = 0.014). However, the subgroup with high hs-CRP (>2 mg/L) had an aHR of 17.51 (95% CI: 2.28–134.52, P = 0.006). The Kaplan-Meier curves for these subgroups were observed in **Figure 6C**.

##### 3.3.2.4 Combined effect with D-Dimer

A high D-Dimer level was associated with higher MACE risk in Hcy-BMI-D-Dimer strata (**Figure 5**). Focusing on obese individuals with HHcy, we observed a rise in risk: the aHR for MACE increased from 5.20 (95% CI: 2.18–12.40, P < 0.001) among those with normal D-Dimer to 9.91 (95% CI: 3.68–26.66, P < 0.001) in the high D-Dimer cohort. In the subgroup with high Hcy and BMI < 24 kg/m², the aHR was 4.03 (95% CI: 1.72–9.44, P=0.001) for normal D-Dimer and 2.86 (95% CI: 0.89–9.17, P=0.078) for high D-Dimer. The corresponding Kaplan-Meier survival curves for these D-Dimer subgroups were presented in **Figure 6D**.

### 3.4 Sensitivity analysis

We calculated the E-value for our primary finding to assess the potential impact of unmeasured confounding. The observed adjusted HR of 2.72 for high Hcy corresponds to an E-value of 5.15. This indicates that, to fully explain this association, the unmeasured confounding factors would need to have a relative risk of at least 5.15 for both high Hcy and MACE, and such an association would have to exceed the influence of the already adjusted covariates. The existence of such a strong single confounder is unlikely, which strengthens the robustness of our findings.

## 4 Discussion

This study establishes plasma Hcy as an independent prognostic marker for MACE in patients with PMI. This finding is in line with earlier studies showing the important function of Hcy in cardiovascular prognosis (9, 10). Further analysis of the individual endpoints comprising MACE revealed that higher Hcy levels were associated with non-fatal myocardial infarction, rehospitalization for unstable angina, rehospitalization for severe heart failure, and target lesion revascularization (TLR). Stroke and cardiac death also showed similar trends, but the differences were not statistically significant. Given the low number of events for some endpoints, these results should be interpreted with caution. Moreover, our findings suggest that elevated Hcy serves as a marker for systemic metabolic homeostasis disruption. Recent analyses have reinforced this, showing that the Hcy-CAD association is particularly robust in Asian populations, likely driven by the unique genetic and dietary interactions characterizing this cohort (22). Even though our observations align with numerous established studies, the prognostic significance of Hcy has long been controversial. The disappointing neutral outcomes observed in initial, extensive RCTs of Hcy-lowering B vitamins were a major contributing factor to this controversy (23). However, from an endocrine perspective, these “failures” likely stem from a lack of metabolic phenotyping rather than the molecule’s irrelevance. Previous trials largely adopted a non-stratified approach, ignoring the complex interactions between Hcy and host metabolic status (e.g., adiposity and insulin resistance). By failing to isolate the specific “high-risk metabolic phenotypes” where Hcy acts as a risk marker, these studies may have diluted the potential therapeutic benefits. Our study directly addresses this gap and proposes a transformative clinical concept: Hcy functions as a critical “risk marker”, acting not in a vacuum, but as a marker associated with the underlying “metaflammation” that defines the PMI phenotype (1, 2). In this well-established pro-inflammatory context—already fueled by cytokines from inflamed adipose tissue—our data reveal that Hcy may be more than a bystander. This association appeared strongest among patients with obesity. The aHR for MACE in obese patients with high Hcy reached 6.54 (95% CI, 3.01-14.20,P<0.001) compared to the low-risk reference. This is consistent with metabolomic research showing that HHcy, particularly in obese people, has been linked to coronary artery pathology (24).

The biochemical basis of this combined association is a vicious loop in which adipose tissue-driven “metaflammation” may be aggravated by Hcy. This happens through several pathways relevant to metabolic homeostasis: Initially, it intensifies inflammation at its origin. While increased cytokine release from obese adipose tissue produces a pro-inflammatory environment (2), Hcy acts as an accelerant by activating critical innate immune pathways, with a particular emphasis on the NLRP3 inflammasome, leading to mature IL-1β production and escalating atherogenesis (4, 25). Second, Hcy induces a profound endocrine dysfunction within the adipose tissue. Beyond merely promoting inflammation, Hcy acts as a metabolic disruptor that suppresses the production of adiponectin, a potent cardioprotective and insulin-sensitizing hormone. Early mechanistic studies established that Hcy-induced endoplasmic reticulum (ER) stress inhibits adiponectin secretion (26). Crucially, recent evidence highlights that this dysfunction is particularly severe in perivascular adipose tissue (PVAT), where Hcy downregulates adiponectin receptors and impairs the eNOS signaling pathway, thereby potentially weakening local vascular protection (27). Furthermore, it acts as a potent disruptor of systemic insulin responsiveness. HHcy physically modifies the insulin receptor precursor (pro-IR) via cysteine-homocysteinylation (C-Hcy). This modification interferes with Furin protease cleavage, thereby blocking the maturation of the insulin receptor and blunting systemic insulin signaling at the molecular level (28). In addition, Hcy induces ER stress in adipose tissue, which activates inflammatory pathways (JNK, NF-κB) known to drive systemic IR (28, 29). Finally, it has been associated with abnormalities in lipid metabolism. Hepatic steatosis and atherogenic dyslipidemia are fueled by the flood of free fatty acids released when adipocyte lipolysis is activated (via HIF1α-ERO1α) as a result of this Hcy-driven aggravation (30). Mechanistically, this is exacerbated by an elevated SAH/SAM ratio, which induces hypermethylation of the mitochondrial GPx1 promoter. This epigenetic silencing impairs the antioxidant defense, leading to unchecked oxidative stress (6). Overall, Hcy shifts adipose tissue toward a more pathogenic phenotype. However, obesity is not a homogeneous condition. Prior studies have shown that central adiposity is more strongly associated with adverse cardiovascular outcomes and metabolic dysfunction (31). More recent data also indicate that visceral adiposity is linked to a higher risk of major cardiovascular events and mortality compared with peripheral fat distribution (32). Therefore, we speculate that heterogeneity in obesity may influence Hcy-related risk stratification, and the interaction between different fat distribution phenotypes and Hcy warrants further investigation in future studies.

This association extended beyond just obesity. It may identify a higher-risk clinical setting, allowing inflammation and clotting factors to do far more damage than they normally would. The combination of high Hcy with high Lp(a) (aHR=3.44; 95% CI, 2.07-5.73, P<0.001) suggested a higher-risk profile of both pro-atherogenic and pro-thrombotic tendencies. The pathological process may be inferred: one side, by competing with plasminogen, Lp(a) prevents the body from breaking up clots. On the other side, Hcy chemically modifies fibrinogen through its metabolite, homocysteine-thiolactone. This structural modification renders the thrombus resistant to fibrinolysis (8, 33), potentially contributing to a hypercoagulable state. This pro-thrombotic effect was further underscored by the association with elevated D-Dimer (aHR=3.13; 95% CI,1.85-5.31, P<0.001) (8). Finally, the model suggested that these pathways may collectively contribute to inflammation-related processes, which was also reflected to some extent in individuals with elevated hs-CRP levels (aHR=4.12; 95% CI, 1.93-8.81, P<0.001). In this setting, Hcy may be involved in inflammatory processes, activating pathways like NF-κB (7).

We conducted an exploratory three-factor analysis with the aim of exploring the possible ways by which multiple factors may lead to a high health risk; also to examine in what order Hcy may become more harmful as an amplification factor. The number of patients and MACE events in several of the subgroups was relatively small. The results should be interpreted with caution (**Supplementary Table 5**). The Hcy-obesity-hypertension framework suggested that there was a risk progression among individuals with all three factors present; The hazard ratio was 5.75 times higher than that in those without these conditions (aHR = 5.75; 95% CI, 2.27-14.56; P < 0.001). This pattern may reflect the contribution of obesity and hypertension to the metabolic disturbances underlying hyperhomocysteinemia. The Hcy-obesity-Lp(a) framework showed that a more powerful pro-thrombotic and pro-atherogenic effect was present, and the triple-positive group had 5.93 times the risk (aHR=5.93; 95% CI: 2.32-15.16, P<0.001). This pattern may reflect a more adverse biological profile. Hcy and obesity initially damage the vascular microenvironment; Against this weak link, high levels of Lp(a) and changed fibrinogen cooperate to exceed the body’s own anticoagulation system. The highest risk estimate was observed in the Hcy-obesity-D-Dimer model. The triple-positive group had almost a 10-times higher risk (aHR=9.91; 95% CI:3.68-26.66, P<0.001). This was the final evidence of the perilous outcomes resulting from such connections. According to the hs-CRP model’s research results, inflammation appeared to be an important driver of this pattern. The subjects of the ‘triple-hit’ group had a significantly poorer outcome for MACE (aHR = 17.51; 95% CI: 2.28-134.52, P = 0.006). Mechanistically, this is highly plausible. Hcy promotes inflammation caused by obesity through the activation of the NLRP3 inflammasome complex, for example. This may significantly increase the risk of vascular damage at a high level of hs-CRP (4, 25).

It can be said that there is a possible biological relevance; however, in view of the occurrence of “treatment masking” and high variability in some small subgroups, we are more cautious when addressing these associations among them. Subjects with elevated Hcy levels and blood pressure, particularly when they were ‘elevated’, exhibited a significantly higher risk of adverse outcomes than those in the ‘high’ blood pressure group (aHR=3.28; 95% CI: 1.60-6.74; P = 0.001) and aHR=2.63; 95% CI: 1.26-5.47; P = 0.010). **Supplementary Table 2** showed that subjects in the ‘high’ blood pressure group tended to be treated more intensively for cardiovascular prevention, such as the increased use of ACEI/ARBs and beta-blockers, which may have reduced their long-term risk. In a tripartite model, for instance, in the Hcy-obesity-hs-CRP framework, the hs-CRP low group actually had a higher risk score (aHR = 21.04), but it is still affected by large confidence intervals due to insufficient data; therefore, it cannot be considered reliable. Information in **Supplementary Table 3** showed that the weight of individuals with increased hs-CRP and Hcy was greater than that of other groups; therefore, rigorous clinical intervention (statin use and ACEI/ARB prescription) was given to these people, which might have concealed this group’s true risk. We observed a similar trend for the Hcy-BMI-D-Dimer combined pattern. Among those with high levels of Hcy and normal BMI, there was no significant difference in the category of elevated D-Dimer (>0.5 mg/L) compared with its low-dimer counterpart (<0.5 mg/L) (aHR = 2.86, 95% CI: 0.89-9.17, P = 0.078), and their baseline characteristics were presented in **Supplementary Table 4**. In short, although supporting the ‘multiple hits’ approach, these risk estimates need to be evaluated with caution. Although the above findings suggest new hypotheses, they require further verification in more extensive populations. There was insufficient evidence of sophisticated modeling or of a rigorous three-way analysis.

Homocysteine plays a pivotal role in the one-carbon metabolism pathway, and its levels are closely associated with nutritional factors such as folate, vitamin B6, vitamin B12, and betaine. Traditional Chinese dietary habits have led to a significantly higher prevalence of elevated homocysteine levels among the Chinese population compared to Western countries. Existing systematic reviews and meta-analyses suggest that supplementation with folate, B vitamins, and betaine may reduce homocysteine levels to some extent (34–36). The Chinese population is more prone to elevated homocysteine levels or hyperhomocysteinemia, a phenomenon that may be related to factors such as genetic background and dietary patterns. The core intervention for high homocysteine levels involves combined supplementation with B vitamins; for cases of persistent elevation, betaine may be added (37). However, the results of our study are not yet sufficient to directly propose specific precision nutrition intervention strategies.

### 4.1 Limitations

Although this study has a number of clinical implications, we also need to recognize its limits.

At present, there is an initial problem of potential attrition bias in this study. Seventy participants who dropped out did not have cardiac performance data or increased inflammatory biomarker levels, but they had decreased Hcy concentrations. Excluding the particular group of “high-risk, low-Hcy” might lead to a high correlation between elevated Hcy and MACE being overestimated. Secondly, this study used clinical data from only one hospital in Tianjin, China. Subsequent multicenter, prospective trials that integrate Mendelian randomization methods may effectively strengthen the current body of evidence on causal connections for Hcy. Finally, it was not assessed whether B-vitamin status, such as folate and B12/B6, or essential genetic polymorphisms (MTHFR C677T) that serve as primary regulators of Hcy were present in the subjects; these elements would have added additional weight to the results. Inclusion of these elements will help us better understand the Hcy metabolic pathway and identify nutritional and genetic causes of HHcy. These variables should be incorporated in future studies to improve the precision of Medical Nutrition Therapy (MNT) recommendations. Additionally, in the future, it may be examined whether all kinds of Hcy derivatives (e.g., Hcy-thiolactone) have a significant effect on PMI; thus, new biomarkers and treatment target molecules can also emerge. Previous studies have shown that some degradation products of Hcy, such as Hcy-thiolactone and AdoHcy, may be more closely related to vascular damage than total Hcy; therefore, the determination of these metabolites is expected to enhance risk evaluation (25).

## 5 Conclusion

Our study establishes that in PMI patients, Hcy is more than just an independent predictor of long-term heart problems (MACE). It also acts as a powerful metabolic ‘risk marker.’ Specifically, it worsens the prognosis when combined with other metabolic risks, especially obesity and inflammation. Based on this, incorporating Hcy in routine metabolic assessments may be clinically significant and help achieve more precise risk stratification, particularly in patients with elevated Hcy levels and concomitant metabolic abnormalities such as obesity.

## Data Availability

The original contributions presented in the study are included in the article/**Supplementary Material**. Further inquiries can be directed to the corresponding authors.
