# Supplementary material for: Elevated homocysteine associated with higher cardiovascular risk in young patients with myocardial infarction: the role of metabolic phenotypes
Source: Front Endocrinol (Lausanne). 2026 May 8;17:1821132. doi: 10.3389/fendo.2026.1821132 (PMC13193844; doi:10.3389/fendo.2026.1821132)
Supplement: Supplementary file 1 [file DataSheet1.docx]

Supplementary Material

1. Supplementary Figure


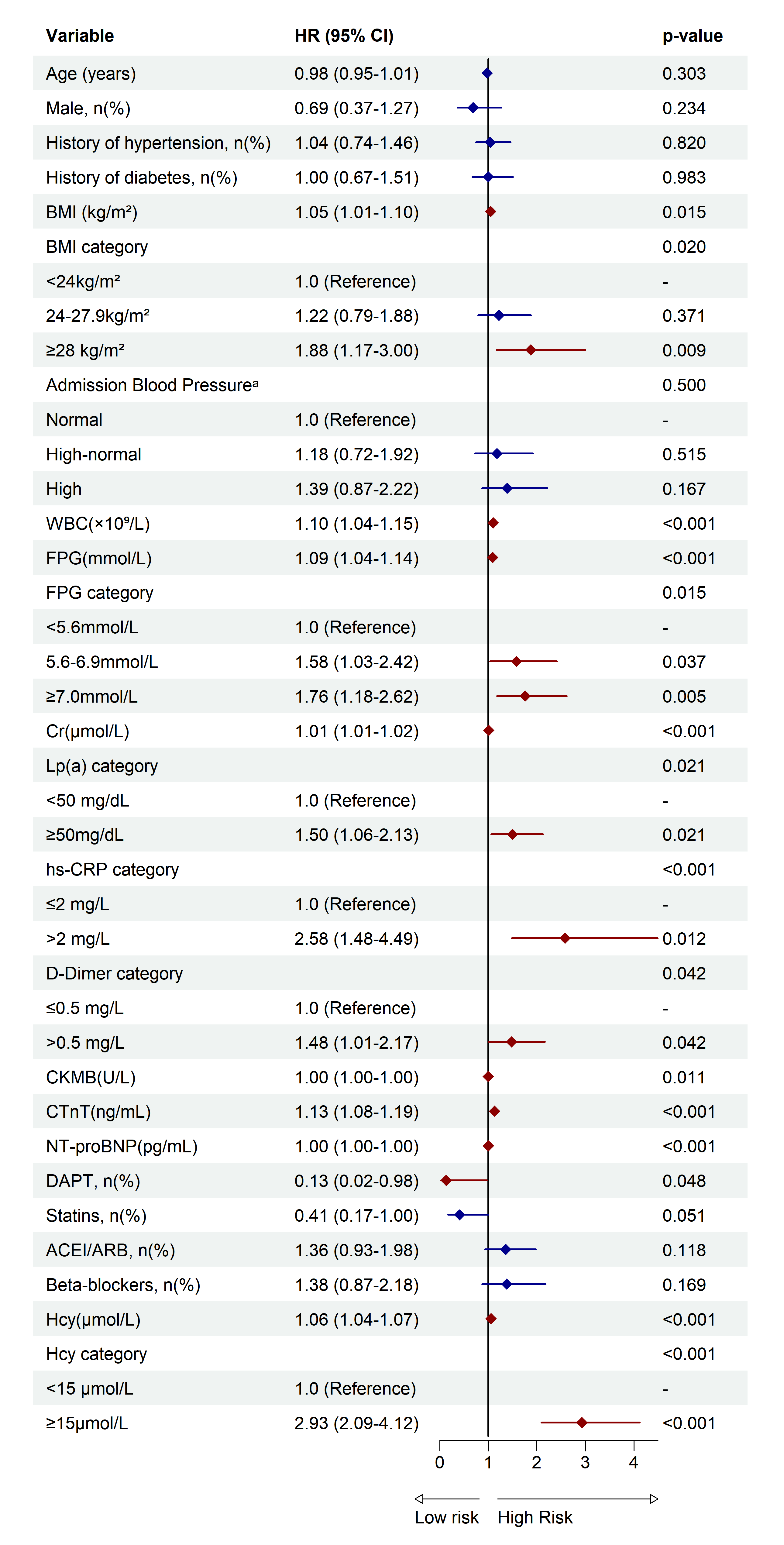


Supplemental Figure 1 Univariate Cox Regression Analysis of MACE Risk Factors

^a^Admission blood pressure:Normal:the systolic blood pressure (SBP) <120 mmHg and the diastolic blood pressure (DBP) is <80 mmHg; Normal-High:the SBP is between 120 to139 mmHg and/or the DBP is between 80 to 89 mmHg; High:the SBP ≥140 mmHg and/or the DBP ≥90 mmHg.

Abbreviations are the same as those defined in Table 1.

1. Supplementary Tables

| **Supplemental Table S1. Baseline Characteristics of Patients Stratified by Follow-up Status** | | | | |
| --- | --- | --- | --- | --- |
| **Variables** | **Total Population (N = 1290)** | **Follow-up**  **(N = 1220)** | **Lost to Follow-up**  **(N = 70)** | **P-value** |
| **Baseline Demographics** |  |  |  |  |
| Age (years) | 41.00 (37.00–44.00) | 41.00 (37.00–44.00) | 42.50 (38.00–48.00) | 0.004 |
| Male, n (%) | 1,130 (87.60) | 1,082 (88.69) | 48.00 (68.57) | <0.001 |
| BMI (kg/m²) | 26.00 (23.90–28.30) | 26.00 (23.90–28.10) | 26.00 (23.40–30.10) | 0.821 |
| BMI category, n (%) |  |  |  | 0.062 |
| <24kg/m² | 329 (25.50) | 308 (25.25) | 21 (30.00) |  |
| 24–27.9kg/m² | 615 (47.67) | 591 (48.44) | 24 (34.29) |  |
| ≥28 kg/m² | 346 (26.82) | 321 (26.31) | 25 (35.71) |  |
| Admission blood pressure, n (%)^a^ |  |  |  | 0.939 |
| Normal | 254 (19.69) | 240 (19.67) | 14 (20.00) |  |
| High-normal | 486 (37.67) | 461 (37.79) | 25 (35.71) |  |
| High | 550 (42.64) | 519 (42.54) | 31 (44.29) |  |
| History of smoking, n (%) | 798 (61.86) | 761 (62.38) | 37 (52.86) | 0.111 |
| History of alcohol consumption, n (%) | 420 (32.56) | 398 (32.62) | 22 (31.43) | 0.836 |
| **Medical History** |  |  |  |  |
| History of hypertension, n (%) | 629 (48.76) | 586 (48.03) | 43 (61.43) | 0.029 |
| History of diabetes, n (%) | 267 (20.70) | 252 (20.66) | 15 (21.43) | 0.877 |
| History of chronic kidney disease, n (%) | 15 (1.16) | 15 (1.23) | 0 (0.00) | >0.999 |
| History of cerebrovascular disease, n (%) | 45 (3.49) | 41 (3.36) | 4 (5.71) | 0.302 |
| Prior angina, n (%) | 172 (13.33) | 170 (13.93) | 2 (2.86) | 0.008 |
| Prior myocardial infarction, n (%) | 70 (5.43) | 68 (5.57) | 2 (2.86) | 0.582 |
| Prior coronary artery disease, n (%) | 126 (9.77) | 126 (10.33) | 0 (0.00) | 0.005 |
| Prior PCI, n (%) | 48 (3.72) | 47 (3.85) | 1 (1.43) | 0.513 |
| Prior CABG, n (%) | 8 (0.62) | 8 (0.66) | 0 (0.00) | >0.999 |
| **Blood Routine and Inflammatory Indicators** |  |  |  |  |
| WBC (10⁹/L) | 10.20 (8.39–12.35) | 10.17 (8.38–12.30) | 10.63 (8.72–12.61) | 0.146 |
| NEUT (%) | 73.55 (66.50–80.00) | 73.50 (66.50–79.90) | 74.55 (68.90–81.50) | 0.328 |
| RBC (10¹²/L) | 4.85 (4.48–5.17) | 4.85 (4.49–5.18) | 4.75 (4.30–5.05) | 0.059 |
| Hb (g/L) | 147.00 (137.00–157.00) | 147.00 (137.00–157.00) | 143.50 (128.00–158.00) | 0.09 |
| PLT (10⁹/L) | 242.50 (208.00–284.00) | 241.00 (208.00–283.00) | 254.50 (212.00–290.00) | 0.166 |
| hs-CRP (mg/L) | 5.37 (2.40–11.76) | 5.34 (2.38–11.55) | 6.42 (2.76–15.51) | 0.221 |
| hs-CRP category, n (%) |  |  |  | 0.359 |
| ≤2 mg/L | 603 (46.74) | 574 (47.05) | 29 (41.43) |  |
| >2mg/L | 687 (53.26) | 646 (52.95) | 41 (58.57) |  |
| **Biochemical Parameters** |  |  |  |  |
| HbA1c (%) | 5.80 (5.40–7.20) | 5.80 (5.40–7.20) | 5.95 (5.50–8.70) | 0.258 |
| FPG (mmol/L) | 5.79 (5.06–7.60) | 5.77 (5.06–7.51) | 6.20 (5.13–9.36) | 0.082 |
| FPG category, n (%) |  |  |  | 0.3 |
| <5.6mmol/L | 560 (43.41) | 534 (43.77) | 26 (37.14) |  |
| 5.6–6.9mmol/L | 338 (26.20) | 321 (26.31) | 17 (24.29) |  |
| ≥7.0mmol/L | 392 (30.39) | 365 (29.92) | 27 (38.57) |  |
| BUN (mmol/L) | 4.30 (3.60–5.30) | 4.30 (3.60–5.30) | 4.20 (3.50–5.80) | 0.557 |
| Cr (μmol/L) | 73.00 (64.00–83.00) | 73.00 (64.00–83.00) | 74.00 (61.00–86.00) | 0.946 |
| Uric acid (μmol/L) | 356.50 (295.00–428.00) | 356.00 (295.50–427.00) | 368.00 (284.00–437.00) | 0.918 |
| TBIL (μmol/L) | 14.00 (10.06–19.10) | 13.96 (10.00–19.00) | 14.88 (11.20–21.50) | 0.126 |
| DBIL (μmol/L) | 4.20 (2.60–6.00) | 4.20 (2.60–5.90) | 4.95 (3.40–7.10) | 0.019 |
| ALT (U/L) | 42.45 (27.30–67.50) | 42.35 (27.20–68.05) | 48.20 (30.10–64.10) | 0.444 |
| AST (U/L) | 104.60 (42.50–210.70) | 103.60 (42.35–208.30) | 120.90 (49.60–249.10) | 0.337 |
| ALP (U/L) | 75.00 (63.00–90.00) | 75.00 (63.00–90.00) | 77.50 (65.00–94.00) | 0.131 |
| GGT (U/L) | 37.55 (25.00–57.00) | 37.80 (25.00–57.05) | 33.95 (24.40–55.90) | 0.557 |
| **Hcy (μmol/L)** | 12.20 (9.51–16.60) | 12.30 (9.61–16.85) | 10.42 (8.07–14.47) | 0.001 |
| **Hcy category, n (%)** |  |  |  | 0.038 |
| <15 μmol/L | 888 (68.84) | 832 (68.20) | 56 (80.00) |  |
| ≥15μmol/L | 402 (31.16) | 388 (31.80) | 14 (20.00) |  |
| **Lipid Profile** |  |  |  |  |
| Lp(a) (mg/dL) | 73.61 (22.50–193.51) | 72.96 (22.55–192.13) | 85.63 (20.20–243.93) | 0.875 |
| Lp(a) category, n (%) |  |  |  | 0.978 |
| <50 mg/dL | 514 (39.84) | 486 (39.84) | 28 (40.00) |  |
| ≥50mg/dL | 776 (60.16) | 734 (60.16) | 42 (60.00) |  |
| TC (mmol/L) | 4.84 (4.12–5.53) | 4.86 (4.14–5.56) | 4.72 (3.98–5.13) | 0.053 |
| TG (mmol/L) | 2.03 (1.45–3.03) | 2.03 (1.45–3.03) | 1.93 (1.45–3.20) | 0.568 |
| HDL-C (mmol/L) | 0.92 (0.82–1.06) | 0.92 (0.82–1.06) | 0.92 (0.81–1.05) | 0.695 |
| LDL-C (mmol/L) | 3.17 (2.48–3.78) | 3.18 (2.47–3.80) | 3.07 (2.56–3.58) | 0.245 |
| VLDL-C (mmol/L) | 0.58 (0.38–0.85) | 0.58 (0.38–0.85) | 0.44 (0.34–0.79) | 0.077 |
| ApoA1 (g/L) | 1.13 (1.00–1.26) | 1.13 (1.00–1.26) | 1.12 (1.03–1.26) | 0.947 |
| ApoB (g/L) | 1.14 (0.93–1.34) | 1.14 (0.93–1.35) | 1.14 (0.95–1.27) | 0.497 |
| Non-HDL-C (mmol/L) | 3.88 (3.15–4.58) | 3.89 (3.16–4.60) | 3.73 (3.05–4.11) | 0.09 |
| **Myocardial Injury Markers** |  |  |  |  |
| CK (U/L) | 981.00 (324.00–2,074.00) | 987.00 (323.50–2,072.50) | 856.00 (331.00–2,200.00) | 0.832 |
| CK-MB (U/L) | 84.00 (31.00–175.00) | 84.00 (30.00–176.05) | 81.00 (40.00–161.00) | 0.514 |
| cTnT (ng/mL) | 1.90 (0.50–4.30) | 1.88 (0.50–4.29) | 2.09 (0.57–4.88) | 0.426 |
| NT-proBNP (pg/mL) | 161.98 (32.48–488.00) | 153.15 (28.86–472.95) | 304.96 (71.32–1,016.00) | <0.001 |
| LVEF (%) | 52.00 (46.00–57.00) | 53.00 (47.00–57.00) | 50.00 (45.00–55.00) | 0.008 |
| **Coagulation** |  |  |  |  |
| D-Dimer (mg/L) | 0.27 (0.19–0.42) | 0.26 (0.19–0.42) | 0.32 (0.21–0.66) | 0.032 |
| D-Dimer category, n (%) |  |  |  | <0.001 |
| ≤0.5 mg/L | 1,047 (81.16) | 1,001 (82.05) | 46 (65.71) |  |
| >0.5 mg/L | 243 (18.84) | 219 (17.95) | 24 (34.29) |  |
| Fibrinogen (g/L) | 3.33 (2.86–3.93) | 3.32 (2.86–3.93) | 3.41 (2.92–3.91) | 0.484 |
| **Clinical and Angiographic Features** |  |  |  |  |
| Diagnosis, n (%) |  |  |  | 0.187 |
| STEMI | 995 (77.13) | 936 (76.72) | 59 (84.29) |  |
| NSTEMI | 295 (22.87) | 284 (23.28) | 11 (15.71) |  |
| **Number of diseased vessels, n (%)** |  |  |  | 0.511 |
| No significant stenosis | 44 (3.41) | 41 (3.36) | 3 (4.29) |  |
| Single-vessel disease | 493 (38.22) | 472 (38.69) | 21 (30.00) |  |
| Double-vessel disease | 345 (26.74) | 326 (26.72) | 19 (27.14) |  |
| Triple-vessel disease | 377 (29.22) | 352 (28.85) | 25 (35.71) |  |
| Left main disease | 31 (2.40) | 29 (2.38) | 2 (2.86) |  |
| Gensini score | 24.00 (0.00–49.00) | 26.00 (0.00–52.00) | 17.00 (0.00–36.00) | 0.07 |
| **Concomitant Medications** |  |  |  |  |
| DAPT, n (%) | 1,285 (99.61) | 1,215 (99.59) | 70 (100.00) | >0.999 |
| Statins, n (%) | 1,259 (97.60) | 1,192 (97.70) | 67 (95.71) | 0.234 |
| ACEI/ARB, n (%) | 861 (66.74) | 806 (66.07) | 55 (78.57) | 0.031 |
| Beta-blocker, n (%) | 1,002 (77.67) | 944 (77.38) | 58 (82.86) | 0.284 |

^a^Admission blood pressure:Normal:the systolic blood pressure (SBP) <120 mmHg and the diastolic blood pressure (DBP) is <80 mmHg; Normal-High:the SBP is between 120 to139 mmHg and/or the DBP is between 80 to 89 mmHg; High:the SBP ≥140 mmHg and/or the DBP ≥90 mmHg

For abbreviations, see footnote to Table 1

| **Supplemental Table S2. Baseline Characteristics by Homocysteine and Admission Blood Pressure** | | | | | | | |
| --- | --- | --- | --- | --- | --- | --- | --- |
| **Variable** | **Normal Hcy, Normal BP**  **(N=157)** | **Normal Hcy, High-Normal BP**  **(N=328)** | **Normal Hcy, Hypertension**  **(N=347)** | **High Hcy, Normal BP**  **(N=83)** | **High Hcy, High-Normal BP**  **(N=133)** | **High Hcy,**  **High BP**  **(N=172)** | **P-value** |
| Age (years) | 42.00 (38.00, 44.00) | 42.00 (37.00, 44.00) | 41.00 (37.00, 44.00) | 41.00 (37.00, 43.00) | 40.00 (36.00, 43.00) | 40.00 (35.75, 43.00) | 0.007 |
| Male, n (%) | 129 (82.2) | 278 (84.8) | 303 (87.3) | 81 (97.6) | 130 (97.7) | 161 (93.6) | <0.001 |
| BMI (kg/m²) | 25.70 (23.50, 27.70) | 26.00 (23.90, 27.70) | 26.10 (24.20, 28.95) | 25.70 (23.55, 27.65) | 25.70 (24.30, 27.80) | 26.25 (24.20, 28.75) | 0.071 |
| History of smoking, n (%) | 98 (62.4) | 177 (54.0) | 212 (61.1) | 65 (78.3) | 94 (70.7) | 115 (66.9) | <0.001 |
| History of alcohol consumption, n (%) | 45 (28.7) | 90 (27.4) | 112 (32.3) | 37 (44.6) | 50 (37.6) | 64 (37.2) | 0.018 |
| History of hypertension, n (%) | 54 (34.4) | 135 (41.2) | 210 (60.5) | 34 (41.0) | 51 (38.3) | 102 (59.3) | <0.001 |
| History of diabetes, n (%) | 36 (22.9) | 82 (25.0) | 86 (24.8) | 11 (13.3) | 11 (8.3) | 26 (15.1) | <0.001 |
| Prior myocardial infarction, n (%) | 9 (5.7) | 16 (4.9) | 23 (6.6) | 1 (1.2) | 11 (8.3) | 8 (4.7) | 0.264 |
| hs-CRP (mg/L) | 5.65 (2.19, 15.28) | 4.78 (2.16, 10.45) | 5.34 (2.37, 11.34) | 5.86 (3.41, 17.38) | 5.74 (2.68, 10.73) | 5.38 (3.00, 12.12) | 0.147 |
| HbA1c (%) | 5.80 (5.40, 7.50) | 5.90 (5.40, 7.60) | 6.00 (5.50, 7.65) | 5.70 (5.50, 6.20) | 5.50 (5.30, 6.10) | 5.70 (5.40, 6.53) | <0.001 |
| FPG (mmol/L) | 5.66 (4.98, 7.83) | 5.87 (5.19, 7.95) | 5.99 (5.13, 8.20) | 5.30 (4.81, 6.22) | 5.48 (4.86, 6.64) | 5.69 (5.05, 7.03) | <0.001 |
| Cr (μmol/L) | 71.00 (62.00, 78.65) | 72.00 (60.13, 80.00) | 71.00 (61.03, 82.00) | 83.00 (74.50, 93.00) | 77.00 (69.00, 84.00) | 77.55 (69.00, 90.25) | <0.001 |
| Uric acid (μmol/L) | 342.00 (268.00, 409.00) | 348.50 (287.75, 422.25) | 350.00 (294.50, 418.50) | 371.00 (313.00, 467.50) | 368.00 (320.00, 433.00) | 380.00 (308.00, 463.00) | <0.001 |
| Lp(a) (mg/dL) | 56.10 (20.20, 155.70) | 77.30 (25.43, 212.20) | 81.30 (25.10, 208.30) | 38.20 (12.25, 115.56) | 69.96 (23.50, 176.20) | 86.62 (28.98, 201.65) | 0.006 |
| LDL-C (mmol/L) | 3.07 (2.43, 3.98) | 3.06 (2.47, 3.66) | 3.23 (2.54, 3.77) | 3.14 (2.44, 3.62) | 3.23 (2.52, 3.92) | 3.25 (2.47, 3.92) | 0.243 |
| VLDL-C (mmol/L) | 0.50 (0.34, 0.74) | 0.60 (0.39, 0.86) | 0.59 (0.38, 0.88) | 0.58 (0.41, 0.88) | 0.61 (0.34, 0.85) | 0.59 (0.40, 0.86) | 0.129 |
| cTnT (ng/mL) | 2.01 (0.70, 4.09) | 1.48 (0.32, 3.80) | 1.76 (0.49, 4.20) | 2.30 (0.74, 5.11) | 2.36 (0.64, 5.69) | 2.58 (0.67, 4.71) | 0.020 |
| NT-proBNP (pg/mL) | 210.32 (46.86, 572.70) | 135.04 (32.47, 362.56) | 104.26 (10.00, 421.55) | 358.50 (94.50, 1159.00) | 221.31 (51.50, 513.20) | 151.13 (11.79, 545.33) | <0.001 |
| D-Dimer(mg/L) | 0.25 (0.19, 0.43) | 0.26 (0.19, 0.39) | 0.27 (0.19, 0.42) | 0.29 (0.21, 0.49) | 0.28 (0.19, 0.46) | 0.25 (0.19, 0.39) | 0.432 |
| LVEF (%) | 52.00 (45.00, 56.00) | 53.50 (48.00, 58.00) | 53.00 (47.00, 57.00) | 50.00 (44.50, 55.00) | 53.00 (48.00, 57.00) | 51.00 (46.00, 56.00) | 0.033 |
| Diagnosis, n (%) 0.105 | | | | | | | |
| STEMI | 124 (79.0) | 236 (72.0) | 276 (79.5) | 67 (80.7) | 107 (80.5) | 126 (73.3) |  |
| NSTEMI | 33 (21.0) | 92 (28.0) | 71 (20.5) | 16 (19.3) | 26 (19.5) | 46 (26.7) |  |
| Concomitant Medications | | | | | | | |
| DAPT, n (%) | 156 (99.4) | 326 (99.4) | 345 (99.4) | 83 (100.0) | 133 (100.0) | 172 (100.0) | 0.948 |
| Statins, n (%) | 155 (98.7) | 322 (98.2) | 337 (97.1) | 81 (97.6) | 130 (97.7) | 167 (97.1) | 0.858 |
| ACEI/ARB, n (%) | 72 (45.9) | 204 (62.2) | 264 (76.1) | 40 (48.2) | 86 (64.7) | 140 (81.4) | <0.001 |
| Beta-blocker, n (%) | 105 (66.9) | 243 (74.1) | 286 (82.4) | 60 (72.3) | 104 (78.2) | 146 (84.9) | <0.001 |

| **Supplemental Table S3. Baseline Characteristics by Homocysteine BMI and hs-CRP** | | | | | | | | | | | | | |
| --- | --- | --- | --- | --- | --- | --- | --- | --- | --- | --- | --- | --- | --- |
| **Variable** | **Hcy<15**μmol/L**, BMI**<24kg/m²**， hs-CRP<2mg/L**  **(N=54)** | **Hcy<15**μmol/L**, BMI**<24kg/m²**，hs-CRP**>2 mg/L  **(N=158)** | **Hcy<15**μmol/L**, BMI** 24–27.9kg/m²**，hs-CRP<2mg/L**  **(N=100)** | **Hcy<15**μmol/L**, BMI** 24–27.9kg/m²**，**  **hs-CRP**>2mg/L**(N=296)** | **Hcy<15**μmol/L**, BMI**≥28 kg/m²**，hs-CRP<2mg/L**  **(N=34)** | **Hcy<15**μmol/L **BMI**≥28 kg/m²**，hs-CRP**>2 mg/L  **(N=190)** | **Hcy**≥15μmol/L**, BMI**<24kg/m²**，hs-CRP<2mg/L**  **(N=25)** | **Hcy**≥15μmol/L**, BMI**<24kg/m²**，hs-CRP**>2mg/L **(N=71)** | **Hcy**≥15μmol/L**, BMI** 24–27.9kg/m²**，hs-CRP<2mg/L**  **(N=39)** | **Hcy**≥15μmol/L**, BMI** 24–27.9kg/m²**，hs-CRP**>2mg/L  **(N=156)** | **Hcy**≥15μmol/L**, BMI**≥28 kg/m²**，hs-CRP<2mg/L**  **(N=12)** | **Hcy**≥15μmol/L**,**  **BMI**≥28 kg/m²**，hs-CRP**>2mg/L  **(N=85)** | **P-value** |
| Age (years) | 43.00 (41.00, 44.75) | 42.00 (37.00, 45.00) | 42.00 (39.00, 44.00) | 42.00 (38.00, 44.00) | 40.50 (38.00, 44.75) | 40.00 (36.00, 43.00) | 44.00 (38.00, 45.00) | 41.00 (36.00, 43.00) | 40.00 (37.50, 44.00) | 40.00 (36.00, 43.00) | 41.50 (37.75, 43.50) | 39.00 (35.00, 43.00) | <0.001 |
| Male, n (%) | 42 (77.8) | 124 (78.5) | 88 (88.0) | 263 (88.9) | 27 (79.4) | 166 (87.4) | 21 (84.0) | 68 (95.8) | 39 (100.0) | 149 (95.5) | 11 (91.7) | 84 (98.8) | <0.001 |
| BMI (kg/m²) | 22.75 (21.42, 23.32) | 22.60 (21.40, 23.40) | 25.70 (25.20, 26.72) | 26.00 (25.20, 26.72) | 31.00 (29.18, 32.40) | 30.90 (28.70, 32.98) | 22.70 (21.00, 23.10) | 22.40 (21.45, 23.10) | 26.00 (25.40, 26.80) | 26.00 (25.20, 27.20) | 30.55 (29.62, 31.25) | 30.90 (29.10, 32.70) | <0.001 |
| History of smoking, n (%) | 34 (63.0) | 86 (54.4) | 55 (55.0) | 184 (62.2) | 23 (67.6) | 105 (55.3) | 14 (56.0) | 55 (77.5) | 26 (66.7) | 111 (71.2) | 9 (75.0) | 59 (69.4) | 0.005 |
| History of alcohol consumption, n (%) | 20 (37.0) | 55 (34.8) | 23 (23.0) | 87 (29.4) | 10 (29.4) | 52 (27.4) | 7 (28.0) | 34 (47.9) | 14 (35.9) | 61 (39.1) | 5 (41.7) | 30 (35.3) | 0.039 |
| History of hypertension, n (%) | 21 (38.9) | 70 (44.3) | 48 (48.0) | 136 (45.9) | 21 (61.8) | 103 (54.2) | 13 (52.0) | 29 (40.8) | 15 (38.5) | 73 (46.8) | 4 (33.3) | 53 (62.4) | 0.047 |
| History of diabetes, n (%) | 14 (25.9) | 50 (31.6) | 17 (17.0) | 79 (26.7) | 4 (11.8) | 40 (21.1) | 2 (8.0) | 17 (23.9) | 5 (12.8) | 15 (9.6) | 1 (8.3) | 8 (9.4) | <0.001 |
| Prior myocardial infarction, n (%) | 1 (1.9) | 7 (4.4) | 8 (8.0) | 14 (4.7) | 5 (14.7) | 13 (6.8) | 0 (0.0) | 5 (7.0) | 2 (5.1) | 9 (5.8) | 0 (0.0) | 4 (4.7) | 0.519 |
| hs-CRP (mg/L) | 0.90 (0.66, 1.23) | 7.15 (3.86, 16.38) | 1.12 (0.69, 1.44) | 7.21 (3.91, 14.05) | 0.99 (0.63, 1.53) | 7.69 (4.30, 14.87) | 1.02 (0.77, 1.39) | 7.42 (4.19, 15.96) | 1.30 (0.85, 1.50) | 7.83 (4.47, 17.70) | 1.06 (0.64, 1.68) | 8.15 (4.67, 16.13) | <0.001 |
| HbA1c (%) | 5.70 (5.30, 6.25) | 6.15 (5.50, 8.50) | 5.80 (5.40, 6.73) | 5.90 (5.50, 7.80) | 5.80 (5.40, 6.22) | 6.10 (5.50, 7.90) | 5.50 (5.30, 6.70) | 5.70 (5.30, 6.60) | 5.50 (5.35, 5.70) | 5.70 (5.40, 6.20) | 5.55 (5.38, 5.90) | 5.70 (5.40, 6.30) | <0.001 |
| FPG (mmol/L) | 5.40 (4.86, 6.60) | 5.88 (5.21, 8.44) | 5.45 (4.72, 6.79) | 5.88 (5.11, 8.60) | 5.61 (5.07, 6.59) | 6.55 (5.42, 8.73) | 5.30 (4.55, 6.30) | 5.71 (5.14, 6.85) | 5.47 (4.84, 6.94) | 5.46 (4.89, 6.34) | 5.87 (5.75, 6.43) | 5.78 (5.08, 7.49) | <0.001 |
| Cr (μmol/L) | 70.00 (60.00, 84.00) | 68.00 (59.00, 77.00) | 73.00 (62.75, 82.25) | 72.42 (62.03, 81.00) | 71.77 (60.40, 79.75) | 70.00 (61.03, 79.00) | 72.00 (68.00, 93.00) | 78.00 (68.50, 92.00) | 76.00 (70.50, 82.00) | 78.97 (71.76, 90.00) | 78.10 (65.08, 88.25) | 77.00 (69.00, 86.64) | <0.001 |
| Uric acid (μmol/L) | 338.00 (274.50, 369.50) | 328.50 (280.75, 397.00) | 351.00 (286.75, 406.25) | 345.00 (280.75, 421.25) | 373.00 (308.50, 418.50) | 363.00 (312.50, 441.75) | 346.00 (297.00, 424.00) | 372.00 (323.50, 449.00) | 400.00 (334.00, 458.00) | 373.50 (308.50, 433.00) | 362.50 (311.50, 438.50) | 383.00 (324.00, 477.00) | <0.001 |
| Lp(a) (mg/dL) | 35.95 (11.35, 183.58) | 56.20 (20.75, 169.58) | 70.53 (24.80, 190.25) | 66.20 (19.73, 166.19) | 122.62 (45.14, 312.18) | 123.66 (44.30, 261.80) | 54.50 (24.90, 143.10) | 82.70 (21.90, 143.35) | 55.28 (21.70, 114.35) | 59.85 (17.58, 155.97) | 150.50 (91.45, 253.98) | 81.81 (17.70, 225.24) | <0.001 |
| LDL-C (mmol/L) | 3.09 (2.37, 3.60) | 3.08 (2.54, 4.00) | 2.82 (2.19, 3.44) | 3.16 (2.56, 3.75) | 3.37 (2.40, 3.90) | 3.29 (2.64, 3.81) | 2.59 (2.38, 3.53) | 3.14 (2.44, 3.83) | 3.25 (2.47, 3.87) | 3.20 (2.41, 3.99) | 3.21 (2.87, 3.37) | 3.34 (2.89, 3.80) | 0.029 |
| VLDL-C (mmol/L) | 0.49 (0.24, 0.69) | 0.61 (0.37, 1.01) | 0.49 (0.32, 0.66) | 0.61 (0.41, 0.92) | 0.48 (0.30, 0.68) | 0.60 (0.43, 0.91) | 0.55 (0.31, 0.79) | 0.65 (0.40, 0.85) | 0.50 (0.26, 0.69) | 0.60 (0.40, 0.86) | 0.43 (0.23, 1.04) | 0.61 (0.40, 0.93) | <0.001 |
| cTnT (ng/mL) | 1.05 (0.32, 2.69) | 1.94 (0.78, 5.14) | 0.51 (0.04, 2.04) | 1.92 (0.60, 4.23) | 0.81 (0.08, 2.47) | 2.04 (0.58, 4.22) | 1.18 (0.22, 2.61) | 3.00 (0.85, 5.67) | 1.57 (0.16, 5.59) | 2.35 (0.80, 5.10) | 1.99 (0.59, 4.15) | 2.70 (0.98, 5.53) | <0.001 |
| NT-proBNP (pg/mL) | 112.40 (40.03, 285.18) | 241.03 (85.80, 662.03) | 79.12 (18.30, 191.88) | 179.09 (46.26, 511.24) | 58.02 (5.30, 146.50) | 71.27 (8.01, 329.68) | 139.30 (53.77, 395.00) | 369.50 (100.55, 991.65) | 86.43 (14.28, 376.40) | 258.95 (76.75, 739.88) | 5.46 (0.78, 20.37) | 162.40 (13.48, 478.90) | <0.001 |
| D-Dimer（mg/L） | 0.22 (0.19, 0.36) | 0.29 (0.22, 0.43) | 0.22 (0.17, 0.31) | 0.26 (0.18, 0.40) | 0.20 (0.17, 0.26) | 0.28 (0.19, 0.47) | 0.21 (0.16, 0.29) | 0.26 (0.21, 0.37) | 0.25 (0.15, 0.37) | 0.30 (0.21, 0.49) | 0.21 (0.13, 0.40) | 0.28 (0.19, 0.46) | <0.001 |
| LVEF (%) | 56.00 (50.00, 59.00) | 52.00 (47.00, 57.00) | 55.00 (49.00, 59.25) | 53.00 (47.00, 57.00) | 54.50 (48.25, 58.00) | 52.00 (46.00, 56.00) | 54.00 (49.00, 59.00) | 50.00 (43.00, 54.00) | 53.00 (46.00, 58.00) | 52.00 (48.00, 56.00) | 50.00 (45.75, 52.75) | 51.00 (47.00, 57.00) | <0.001 |
| Diagnosis, n (%) |  |  |  |  |  |  |  |  |  |  |  |  | 0.107 |
| STEMI | 34 (63.0) | 116 (73.4) | 72 (72.0) | 233 (78.7) | 25 (73.5) | 156 (82.1) | 16 (64.0) | 57 (80.3) | 31 (79.5) | 116 (74.4) | 11 (91.7) | 69 (81.2) |  |
| NSTEMI | 20 (37.0) | 42 (26.6) | 28 (28.0) | 63 (21.3) | 9 (26.5) | 34 (17.9) | 9 (36.0) | 14 (19.7) | 8 (20.5) | 40 (25.6) | 1 (8.3) | 16 (18.8) |  |
| Concomitant Medications |  |  |  |  |  |  |  |  |  |  |  |  |  |
| DAPT, n (%) | 53 (98.1) | 156 (98.7) | 99 (99.0) | 295 (99.7) | 34 (100.0) | 190 (100.0) | 25 (100.0) | 71 (100.0) | 39 (100.0) | 156 (100.0) | 12 (100.0) | 85 (100.0) | 0.446 |
| Statins, n (%) | 52 (96.3) | 156 (98.7) | 96 (96.0) | 291 (98.3) | 34 (100.0) | 185 (97.4) | 25 (100.0) | 70 (98.6) | 39 (100.0) | 154 (98.7) | 9 (75.0) | 81 (95.3) | 0.037 |
| ACEI/ARB, n (%) | 33 (61.1) | 91 (57.6) | 59 (59.0) | 196 (66.2) | 24 (70.6) | 137 (72.1) | 20 (80.0) | 45 (63.4) | 21 (53.8) | 103 (66.0) | 7 (58.3) | 70 (82.4) | 0.003 |
| Beta-blocker, n (%) | 41 (75.9) | 120 (75.9) | 69 (69.0) | 226 (76.4) | 26 (76.5) | 152 (80.0) | 18 (72.0) | 58 (81.7) | 31 (79.5) | 123 (78.8) | 9 (75.0) | 71 (83.5) | 0.664 |

| **Supplemental Table S4. Baseline Characteristics by Homocysteine BMI and D-Dimer** | | | | | | | | | | | | | |
| --- | --- | --- | --- | --- | --- | --- | --- | --- | --- | --- | --- | --- | --- |
| **Variable** | **Hcy<15**μmol/L**, BMI**<24kg/m²**， D-Dimer**≤0.5 mg/L  **(N=176)** | **Hcy<15**μmol/L**, BMI**<24kg/m²**，D-Dimer**>0.5 mg/L  **(N=36)** | **Hcy<15**μmol/L**, BMI** 24–27.9kg/m²**，D-Dimer**≤0.5 mg/L  **(N=337)** | **Hcy<15**μmol/L**, BMI** 24–27.9kg/m²**，**  **D-Dimer**>0.5 mg/L  **(N=59)** | **Hcy<15**μmol/L**, BMI**≥28 kg/m²**，D-Dimer**≤0.5 mg/L  **(N=179)** | **Hcy<15**μmol/L **BMI**≥28 kg/m²**，D-Dimer**>0.5 mg/L  **(N=45)** | **Hcy**≥15μmol/L**, BMI**<24kg/m²**，D-Dimer**≤0.5 mg/L  **(N=79)** | **Hcy**≥15μmol/L**, BMI**<24kg/m²**，D-Dimer**>0.5 mg/L **(N=17)** | **Hcy**≥15μmol/L**, BMI** 24–27.9kg/m²**，D-Dimer**≤0.5 mg/L  **(N=154)** | **Hcy**≥15μmol/L**, BMI** 24–27.9kg/m²,**D-Dimer**>0.5 mg/L  **(N=41)** | **Hcy**≥15μmol/L**, BMI**≥28 kg/m²**，D-Dimer**≤0.5 mg/L  **(N=76)** | **Hcy**≥15μmol/L**,**  **BMI**≥28 kg/m²**，D-Dimer**>0.5 mg/L  **(N=21)** | **P-value** |
| Age (years) | 42.00 (38.00, 45.00) | 41.50 (36.75, 44.25) | 42.00 (38.00, 44.00) | 42.00 (39.00, 44.50) | 40.00 (37.00, 43.50) | 41.00 (34.00, 43.00) | 42.00 (37.00, 44.00) | 39.00 (35.00, 44.00) | 40.00 (36.00, 43.00) | 40.00 (36.00, 44.00) | 39.00 (36.00, 43.00) | 40.00 (36.00, 43.00) | <0.001 |
| Male, n (%) | 142 (80.7) | 24 (66.7) | 299 (88.7) | 52 (88.1) | 152 (84.9) | 41 (91.1) | 72 (91.1) | 17 (100.0) | 149 (96.8) | 39 (95.1) | 74 (97.4) | 21 (100.0) | <0.001 |
| BMI (kg/m²) | 22.60 (21.40, 23.40) | 22.80 (21.75, 23.42) | 26.00 (25.20, 26.70) | 26.00 (25.00, 26.90) | 30.90 (28.70, 32.80) | 30.70 (29.10, 32.90) | 22.30 (21.35, 23.10) | 23.00 (22.40, 23.10) | 26.05 (25.30, 27.00) | 26.00 (25.10, 27.20) | 30.85 (29.17, 32.80) | 30.40 (29.30, 31.70) | <0.001 |
| History of smoking, n (%) | 104 (59.1) | 16 (44.4) | 210 (62.3) | 29 (49.2) | 102 (57.0) | 26 (57.8) | 56 (70.9) | 13 (76.5) | 109 (70.8) | 28 (68.3) | 48 (63.2) | 20 (95.2) | <0.001 |
| History of alcohol consumption, n (%) | 66 (37.5) | 9 (25.0) | 93 (27.6) | 17 (28.8) | 51 (28.5) | 11 (24.4) | 35 (44.3) | 6 (35.3) | 62 (40.3) | 13 (31.7) | 24 (31.6) | 11 (52.4) | 0.019 |
| History of hypertension, n (%) | 72 (40.9) | 19 (52.8) | 161 (47.8) | 23 (39.0) | 103 (57.5) | 21 (46.7) | 34 (43.0) | 8 (47.1) | 69 (44.8) | 19 (46.3) | 45 (59.2) | 12 (57.1) | 0.074 |
| History of diabetes, n (%) | 57 (32.4) | 7 (19.4) | 85 (25.2) | 11 (18.6) | 34 (19.0) | 10 (22.2) | 14 (17.7) | 5 (29.4) | 16 (10.4) | 4 (9.8) | 8 (10.5) | 1 (4.8) | <0.001 |
| Prior myocardial infarction, n (%) | 8 (4.5) | 0 (0.0) | 21 (6.2) | 1 (1.7) | 18 (10.1) | 0 (0.0) | 4 (5.1) | 1 (5.9) | 7 (4.5) | 4 (9.8) | 2 (2.6) | 2 (9.5) | 0.094 |
| hs-CRP (mg/L) | 4.45 (1.77, 11.25) | 5.55 (2.67, 11.55) | 4.26 (1.92, 10.36) | 6.41 (2.95, 15.97) | 5.76 (2.62, 11.23) | 10.50 (5.65, 20.00) | 4.69 (1.57, 9.69) | 8.61 (4.18, 20.00) | 5.44 (2.54, 10.85) | 7.27 (4.18, 18.77) | 6.07 (3.72, 12.21) | 10.02 (3.82, 25.00) | <0.001 |
| HbA1c (%) | 5.95 (5.50, 8.53) | 5.50 (5.30, 6.82) | 5.90 (5.50, 7.40) | 5.80 (5.40, 6.55) | 6.00 (5.50, 7.30) | 6.10 (5.50, 8.30) | 5.60 (5.30, 6.15) | 6.20 (5.40, 8.90) | 5.65 (5.40, 6.07) | 5.80 (5.50, 6.40) | 5.70 (5.38, 6.35) | 5.60 (5.40, 6.20) | <0.001 |
| FPG (mmol/L) | 5.83 (5.14, 8.11) | 5.68 (5.02, 7.49) | 5.70 (4.98, 7.83) | 5.66 (5.18, 7.79) | 6.25 (5.27, 8.04) | 6.83 (5.55, 9.86) | 5.48 (4.82, 6.46) | 6.39 (4.79, 13.14) | 5.42 (4.89, 6.28) | 5.59 (4.86, 6.90) | 5.66 (5.07, 6.65) | 6.74 (5.86, 7.97) | <0.001 |
| Cr (μmol/L) | 70.00 (60.00, 78.00) | 66.37 (54.75, 74.00) | 73.00 (62.04, 82.00) | 71.00 (62.50, 79.50) | 71.00 (61.00, 79.00) | 69.00 (62.00, 78.00) | 74.00 (68.00, 83.00) | 93.00 (85.00, 104.00) | 78.00 (71.00, 88.78) | 81.00 (73.00, 92.00) | 77.55 (67.00, 88.14) | 79.00 (74.00, 84.00) | <0.001 |
| Uric acid (μmol/L) | 333.00 (283.00, 381.25) | 331.00 (265.25, 401.50) | 346.00 (287.00, 409.00) | 338.00 (265.00, 430.50) | 360.00 (310.00, 435.50) | 394.00 (315.00, 463.00) | 354.00 (307.00, 433.50) | 416.00 (341.00, 486.00) | 378.50 (313.25, 433.75) | 365.00 (285.00, 445.00) | 402.50 (333.75, 485.00) | 325.00 (310.00, 364.00) | <0.001 |
| Lp(a) (mg/dL) | 59.10 (15.17, 184.05) | 39.35 (19.68, 114.99) | 69.49 (19.80, 164.20) | 92.49 (23.75, 206.08) | 121.93 (46.85, 266.76) | 128.35 (40.50, 251.26) | 68.80 (24.20, 144.70) | 79.10 (14.80, 129.90) | 70.18 (23.32, 158.39) | 52.30 (13.40, 121.60) | 91.92 (18.24, 203.55) | 92.50 (35.20, 250.30) | <0.001 |
| LDL-C (mmol/L) | 3.13 (2.53, 3.84) | 2.95 (2.16, 3.82) | 3.05 (2.46, 3.60) | 3.41 (2.60, 3.90) | 3.28 (2.59, 3.79) | 3.40 (2.78, 4.26) | 3.06 (2.40, 3.71) | 3.48 (2.53, 3.86) | 3.20 (2.45, 3.92) | 3.35 (2.19, 4.28) | 3.29 (2.89, 3.76) | 3.32 (3.16, 3.95) | 0.145 |
| VLDL-C (mmol/L) | 0.57 (0.33, 0.82) | 0.46 (0.32, 0.73) | 0.57 (0.39, 0.88) | 0.54 (0.38, 0.78) | 0.61 (0.44, 0.89) | 0.47 (0.33, 0.76) | 0.63 (0.36, 0.85) | 0.63 (0.49, 0.83) | 0.60 (0.40, 0.80) | 0.47 (0.27, 0.76) | 0.64 (0.41, 0.96) | 0.48 (0.37, 0.88) | 0.202 |
| cTnT (ng/mL) | 1.48 (0.61, 3.86) | 3.75 (1.39, 5.49) | 1.32 (0.30, 3.41) | 3.13 (1.21, 6.04) | 1.68 (0.44, 3.79) | 3.17 (1.46, 5.50) | 2.29 (0.47, 4.00) | 4.93 (0.56, 6.48) | 1.99 (0.57, 4.54) | 2.55 (1.14, 10.00) | 2.56 (0.65, 4.44) | 3.88 (1.16, 5.94) | <0.001 |
| NT-proBNP (pg/mL) | 209.70 (47.08, 496.53) | 203.53 (102.03, 508.79) | 123.90 (31.37, 381.90) | 255.49 (86.75, 595.86) | 61.54 (6.12, 270.25) | 117.70 (32.48, 465.26) | 284.30 (70.67, 610.15) | 1175.00 (164.00, 1981.00) | 180.00 (36.64, 625.62) | 520.90 (155.40, 916.70) | 83.78 (6.98, 334.22) | 371.10 (37.85, 910.40) | <0.001 |
| D-Dimer（mg/L） | 0.26 (0.19, 0.33) | 0.78 (0.66, 1.08) | 0.22 (0.16, 0.30) | 0.81 (0.60, 1.22) | 0.22 (0.17, 0.31) | 0.68 (0.58, 1.13) | 0.23 (0.18, 0.28) | 0.69 (0.61, 1.37) | 0.25 (0.18, 0.34) | 0.74 (0.62, 1.28) | 0.22 (0.17, 0.32) | 0.72 (0.60, 1.07) | <0.001 |
| LVEF (%) | 55.00 (48.00, 58.00) | 49.50 (45.00, 57.00) | 54.00 (48.00, 58.00) | 48.00 (42.50, 55.50) | 53.00 (46.00, 57.00) | 50.00 (46.00, 55.00) | 51.00 (45.00, 57.00) | 46.00 (40.00, 50.00) | 54.00 (48.00, 57.75) | 49.00 (44.00, 52.00) | 52.00 (47.00, 57.00) | 49.00 (39.50, 53.00) | <0.001 |
| Diagnosis, n (%) |  |  |  |  |  |  |  |  |  |  |  |  | 0.001 |
| STEMI | 121 (68.8) | 29 (80.6) | 255 (75.7) | 50 (84.7) | 139 (77.7) | 42 (93.3) | 57 (72.2) | 16 (94.1) | 109 (70.8) | 38 (92.7) | 62 (81.6) | 18 (85.7) | 121 (68.8) |
| NSTEMI | 55 (31.2) | 7 (19.4) | 82 (24.3) | 9 (15.3) | 40 (22.3) | 3 (6.7) | 22 (27.8) | 1 (5.9) | 45 (29.2) | 3 (7.3) | 14 (18.4) | 3 (14.3) | 55 (31.2) |
|  |  |  |  |  |  |  |  |  |  |  |  |  |  |
| Concomitant Medications |  |  |  |  |  |  |  |  |  |  |  |  |  |
| DAPT, n (%) | 175 (99.4) | 34 (94.4) | 335 (99.4) | 59 (100.0) | 179 (100.0) | 45 (100.0) | 79 (100.0) | 17 (100.0) | 154 (100.0) | 41 (100.0) | 76 (100.0) | 21 (100.0) | 0.173 |
| Statins, n (%) | 172 (97.7) | 36 (100.0) | 329 (97.6) | 58 (98.3) | 177 (98.9) | 42 (93.3) | 78 (98.7) | 17 (100.0) | 154 (100.0) | 39 (95.1) | 71 (93.4) | 19 (90.5) | 0.015 |
| ACEI/ARB, n (%) | 102 (58.0) | 22 (61.1) | 223 (66.2) | 32 (54.2) | 129 (72.1) | 32 (71.1) | 54 (68.4) | 11 (64.7) | 96 (62.3) | 28 (68.3) | 57 (75.0) | 20 (95.2) | 0.011 |
| Beta-blocker, n (%) | 131 (74.4) | 30 (83.3) | 248 (73.6) | 47 (79.7) | 144 (80.4) | 34 (75.6) | 60 (75.9) | 16 (94.1) | 123 (79.9) | 31 (75.6) | 63 (82.9) | 17 (81.0) | 0.528 |

| **Supplemental Table S5. Number of MACE events in each subgroup of the three-way analyses** | | | | |
| --- | --- | --- | --- | --- |
| **Subgroups** | **Hcy** | **BMI** | **additional cardiovascular risk factors** | **Events/N (%)** |
| Hcy + Hypertension + BMI | Hcy <15 µmol/L | BMI <24 kg/m² | No Hypertension | 7/121 (5.8%) |
| Hcy + Hypertension + BMI | Hcy <15 µmol/L | BMI 24–27.9 kg/m² | No Hypertension | 18/212 (8.5%) |
| Hcy + Hypertension + BMI | Hcy <15 µmol/L | BMI ≥28 kg/m² | No Hypertension | 7/100 (7%) |
| Hcy + Hypertension + BMI | Hcy <15 µmol/L | BMI <24 kg/m² | Hypertension | 4/91 (4.4%) |
| Hcy + Hypertension + BMI | Hcy <15 µmol/L | BMI 24–27.9 kg/m² | Hypertension | 12/184 (6.5%) |
| Hcy + Hypertension + BMI | Hcy <15 µmol/L | BMI ≥28 kg/m² | Hypertension | 10/124 (8.1%) |
| Hcy + Hypertension + BMI | Hcy ≥15 µmol/L | BMI <24 kg/m² | No Hypertension | 10/54 (18.5%) |
| Hcy + Hypertension + BMI | Hcy ≥15 µmol/L | BMI 24–27.9 kg/m² | No Hypertension | 18/107 (16.8%) |
| Hcy + Hypertension + BMI | Hcy ≥15 µmol/L | BMI ≥28 kg/m² | No Hypertension | 7/40 (17.5%) |
| Hcy + Hypertension + BMI | Hcy ≥15 µmol/L | BMI <24 kg/m² | Hypertension | 9/42 (21.4%) |
| Hcy + Hypertension + BMI | Hcy ≥15 µmol/L | BMI 24–27.9 kg/m² | Hypertension | 16/88 (18.2%) |
| Hcy + Hypertension + BMI | Hcy ≥15 µmol/L | BMI ≥28 kg/m² | Hypertension | 18/57 (31.6%) |
| Hcy + Lp(a) + BMI | Hcy <15 µmol/L | BMI <24 kg/m² | Lp(a) <50 mg/dL | 8/98 (8.2%) |
| Hcy + Lp(a) + BMI | Hcy <15 µmol/L | BMI 24–27.9 kg/m² | Lp(a) <50 mg/dL | 13/166 (7.8%) |
| Hcy + Lp(a) + BMI | Hcy <15 µmol/L | BMI ≥28 kg/m² | Lp(a) <50 mg/dL | 7/59 (11.9%) |
| Hcy + Lp(a) + BMI | Hcy <15 µmol/L | BMI <24 kg/m² | Lp(a) ≥50 mg/dL | 3/114 (2.6%) |
| Hcy + Lp(a) + BMI | Hcy <15 µmol/L | BMI 24–27.9 kg/m² | Lp(a) ≥50 mg/dL | 17/230 (7.4%) |
| Hcy + Lp(a) + BMI | Hcy <15 µmol/L | BMI ≥28 kg/m² | Lp(a) ≥50 mg/dL | 10/165 (6.1%) |
| Hcy + Lp(a) + BMI | Hcy ≥15 µmol/L | BMI <24 kg/m² | Lp(a) <50 mg/dL | 5/41 (12.2%) |
| Hcy + Lp(a) + BMI | Hcy ≥15 µmol/L | BMI 24–27.9 kg/m² | Lp(a) <50 mg/dL | 15/85 (17.6%) |
| Hcy + Lp(a) + BMI | Hcy ≥15 µmol/L | BMI ≥28 kg/m² | Lp(a) <50 mg/dL | 11/37 (29.7%) |
| Hcy + Lp(a) + BMI | Hcy ≥15 µmol/L | BMI <24 kg/m² | Lp(a) ≥50 mg/dL | 14/55 (25.5%) |
| Hcy + Lp(a) + BMI | Hcy ≥15 µmol/L | BMI 24–27.9 kg/m² | Lp(a) ≥50 mg/dL | 19/110 (17.3%) |
| Hcy + Lp(a) + BMI | Hcy ≥15 µmol/L | BMI ≥28 kg/m² | Lp(a) ≥50 mg/dL | 14/60 (23.3%) |
| Hcy + BMI + hs-CRP | Hcy <15 µmol/L | BMI <24 kg/m² | hs-CRP ≤2 mg/L | 1/54 (1.9%) |
| Hcy + BMI + hs-CRP | Hcy <15 µmol/L | BMI <24 kg/m² | hs-CRP >2 mg/L | 10/158 (6.3%) |
| Hcy + BMI + hs-CRP | Hcy <15 µmol/L | BMI 24–27.9 kg/m² | hs-CRP ≤2 mg/L | 6/100 (6%) |
| Hcy + BMI + hs-CRP | Hcy <15 µmol/L | BMI 24–27.9 kg/m² | hs-CRP >2 mg/L | 24/296 (8.1%) |
| Hcy + BMI + hs-CRP | Hcy <15 µmol/L | BMI ≥28 kg/m² | hs-CRP ≤2 mg/L | 1/34 (2.9%) |
| Hcy + BMI + hs-CRP | Hcy <15 µmol/L | BMI ≥28 kg/m² | hs-CRP >2 mg/L | 16/190 (8.4%) |
| Hcy + BMI + hs-CRP | Hcy ≥15 µmol/L | BMI <24 kg/m² | hs-CRP ≤2 mg/L | 1/25 (4%) |
| Hcy + BMI + hs-CRP | Hcy ≥15 µmol/L | BMI <24 kg/m² | hs-CRP >2 mg/L | 18/71 (25.4%) |
| Hcy + BMI + hs-CRP | Hcy ≥15 µmol/L | BMI 24–27.9 kg/m² | hs-CRP ≤2 mg/L | 3/39 (7.7%) |
| Hcy + BMI + hs-CRP | Hcy ≥15 µmol/L | BMI 24–27.9 kg/m² | hs-CRP >2 mg/L | 31/156 (19.9%) |
| Hcy + BMI + hs-CRP | Hcy ≥15 µmol/L | BMI ≥28 kg/m² | hs-CRP ≤2 mg/L | 2/12 (16.7%) |
| Hcy + BMI + hs-CRP | Hcy ≥15 µmol/L | BMI ≥28 kg/m² | hs-CRP >2 mg/L | 23/85 (27.1%) |
| Hcy + BMI + D-dimer | Hcy <15 µmol/L | BMI <24 kg/m² | D-Dimer ≤0.5 mg/L | 9/176 (5.1%) |
| Hcy + BMI + D-dimer | Hcy <15 µmol/L | BMI <24 kg/m² | D-Dimer >0.5 mg/L | 2/36 (5.6%) |
| Hcy + BMI + D-dimer | Hcy <15 µmol/L | BMI 24–27.9 kg/m² | D-Dimer ≤0.5 mg/L | 26/337 (7.7%) |
| Hcy + BMI + D-dimer | Hcy <15 µmol/L | BMI 24–27.9 kg/m² | D-Dimer >0.5 mg/L | 4/59 (6.8%) |
| Hcy + BMI + D-dimer | Hcy <15 µmol/L | BMI ≥28 kg/m² | D-Dimer ≤0.5 mg/L | 13/179 (7.3%) |
| Hcy + BMI + D-dimer | Hcy <15 µmol/L | BMI ≥28 kg/m² | D-Dimer >0.5 mg/L | 4/45 (8.9%) |
| Hcy + BMI + D-dimer | Hcy ≥15 µmol/L | BMI <24 kg/m² | D-Dimer ≤0.5 mg/L | 14/79 (17.7%) |
| Hcy + BMI + D-dimer | Hcy ≥15 µmol/L | BMI <24 kg/m² | D-Dimer >0.5 mg/L | 5/17 (29.4%) |
| Hcy + BMI + D-dimer | Hcy ≥15 µmol/L | BMI 24–27.9 kg/m² | D-Dimer ≤0.5 mg/L | 22/154 (14.3%) |
| Hcy + BMI + D-dimer | Hcy ≥15 µmol/L | BMI 24–27.9 kg/m² | D-Dimer >0.5 mg/L | 12/41 (29.3%) |
| Hcy + BMI + D-dimer | Hcy ≥15 µmol/L | BMI ≥28 kg/m² | D-Dimer ≤0.5 mg/L | 16/76 (21.1%) |
| Hcy + BMI + D-dimer | Hcy ≥15 µmol/L | BMI ≥28 kg/m² | D-Dimer >0.5 mg/L | 9/21 (42.9%) |
